# Discovering the bacteriome of *Vitis vinifera* cv. Pinot Noir in a conventionally managed vineyard

**DOI:** 10.1038/s41598-020-63154-w

**Published:** 2020-04-15

**Authors:** Elisa Gamalero, Elisa Bona, Giorgia Novello, Lara Boatti, Flavio Mignone, Nadia Massa, Patrizia Cesaro, Graziella Berta, Guido Lingua

**Affiliations:** 1 Università del Piemonte Orientale, Dipartimento di Scienze e Innovazione Tecnologica, Viale T. Michel 11, Alessandria, 15121 Italy; 2 Università del Piemonte Orientale, Dipartimento di Scienze e Innovazione Tecnologica, Piazza San Eusebio 5, 13100 Vercelli, Italy; 3 SmartSeq s.r.l., spin-off of the Università del Piemonte Orientale, Viale T. Michel 11, Alessandria, 15121 Italy

**Keywords:** Microbial ecology, Soil microbiology

## Abstract

The structure of the bacteriome associated with grapevine roots can affect plant development, health and grape quality. We previously investigated the bacterial biodiversity of the *Vitis vinifera* cv. Pinot Noir rhizosphere in a vineyard subjected to integrated pest management. The aim of this work is to characterize the bacteriome of *V. vinifera* cv. Pinot Noir in a conventionally managed vineyard using a metabarcoding approach. Comparisons between the microbial community structure in bulk soil and rhizosphere (variable space) were performed and shifts of bacteriome according to two sampling times (variable time) were characterized. Bacterial biodiversity was higher at the second than at the first sampling and did not differ according to the variable space. Actinobacteria was the dominant class, with *Gaiella* as the most represented genus in all the samples. Among Proteobacteria, the most represented classes were Alpha, Beta and Gamma-Proteobacteria, with higher abundance at the second than at the first sampling time. *Bradyrhizobium* was the most frequent genus among Alpha-Proteobacteria, while *Burkholderia* was the predominant Beta-Proteobacteria. Among Firmicutes, the frequency of *Staphylococcus* was higher than 60% in bulk soil and rhizosphere. Finally, the sampling time can be considered as one of the drivers responsible for the bacteriome variations assessed.

## Introduction

The economic importance of grapevine is undoubted. In 2018, the vine global cultivated lands amounted to 7.4 millions of hectares; five countries represented 50% of the world vineyards: Spain (13%), China (12%), France (12%), Italy (9%) and Turkey (6%). It has been estimated that in 2018 the vine-growing surface area in Italy was 705.000 hectares, with a production of 8.6 million tons of fresh grape^1^. At the global scale, the Pinot Noir cultivated surface area corresponded to 112.000 ha with Germany, Italy and Switzerland as main producer in Europe, and USA, New Zealand and Australia in non-European countries^2^.

Previous research was mainly focused on the characterization of grapevine genome and transcriptome/metabolome with the aim to increase knowledge on the plant responses to the environment and to abiotic and biotic stresses^3–6^. However, both the growth and health of plants are strictly related to the associated microbiota. Indeed, plants should not be considered as single entities, but as a superorganism or a holobiont, resulting from the plant-microorganisms and microbe-microbe interactions^7,8^. In this context, interactions among bacteria and fungi together with physical factors as climate^9^ or soil parameters^10,11^, vineyard age^12^, rootstock genotypes^13^, as well as soil management^14,15^ and oenological processing^16^, contribute to the determination of a specific terroir^17^. The concept of terroir is based on wine sensory and organoleptic features related to the geographic origin^18^ and is defined by the interactions of plants with environmental and human factors^19^. The role of microorganisms associated to vine in defining the terroir has been ignored for a long time, except for plant pathogens, mainly for the unavailability of tools allowing to have a complete idea of the bacterial and fungal species associated with plants.

In 2014, Bokulich *et al*.^17^ evaluated the relative abundances of bacteria and fungi in grape must, obtained from plants grown in eight vineyards located in four of the major vine growing regions in California, through next-generation sequencing. The data presented in that work show that the bacterial and fungal microbiota occurring during early fermentation stage differ according to the vine-growing regions, and is affected by the grape variety and the year of production.

On the other hand, the microbiota associated to grapevine is affected by the chemical treatments applied in conventional viticulture; negative effects on soil microbial communities are induced by the use of fungicides^20,21^, by acidification of the soil following the fertilizer application^22,23^, and by pesticide application^24^. Altogether, these practices can result in modification of the dynamic interactions between grapevines and microorganisms.

More recently, the grapevine microbiota has been placed at the center of the investigation aiming at understanding the possible beneficial effects of microorganisms on grape production. Thanks to culture-independent methods, and especially to the recent advances in next-generation sequencing methods, the complexity of the grapevine/rhizosphere microbial community has been deeply explored. In a previous work^25^, we characterized, by a metabarcoding approach, the bacteriome of the roots of *V. vinifera* cv. Pinot Noir, in a vineyard subjected to integrated pest management (IPM), looking at the shifts induced by the plant phenological stage and/or by the presence of the plant itself. The main result of that work was that the bacterial community, dominated by Actinobacteria, Proteobacteria, Gemmatimonadetes and Bacteroidetes, responds more intensely to the rhizosphere effect than to the phenological stage of the plant.

In a further study, we used a metaproteome approach in order to characterize the microbial community associated to this IPM vineyard soil and to the roots of *Vitis vinifera* cv. Pinot Noir not only under a taxonomic perspective, but also under a functional point of view^26^. The results showed that bacteria belonging to the genera *Streptomyces, Bacillus*, *Bradyrhizobium, Burkholderia* and *Pseudomonas*, that were quantitatively not dominant in the grapevine rhizosphere community, in terms of DNA abundance, were the most active in protein expression and especially involved in phosphorus and nitrogen metabolism. With this two studies, one focused on metagenome and the other one on metaproteome of the grapevine bacteriome, we obtained a complete description of “actors” and “roles” involved in plant-microbe interactions in an IPM vineyard.

Since vineyard management practices can alter the soil environment, and thus may contribute to shaping the microbial community, in this work we aim to add another piece of the puzzle and characterize, by a metabarcoding approach, the bacterial communities of the roots of *V. vinifera* cv. Pinot Noir, in a vineyard subjected to conventional management. Attention was focused on the shifts induced by the sampling times corresponding to two phenological stages of the plant (first sampling, flowering, and second sampling, early fruiting stage; variable: time) and/or by the rhizosphere effects itself (comparison between bulk soil and rhizosphere, variable: space).

## Results

### Biodiversity

The biodiversity of the bacterial populations in the rhizosphere and bulk soil at the two sampling times were examined by the rarefaction curves (Fig. S1, supplementary material), that is based on the observation that the curve of rarefied counts of any feature should plateau if the sample is close to saturation^27^ thus providing a measure of the depth of our experiments.

The number of observations was sufficient to obtain a good coverage of the entire community in all the samples.

A total of 221,798 reads were obtained with a mean value of 11,000 reads per sample. After the demultiplexing step, a total of 205,827 reads (with a mean value of 10,290 reads per sample) were used for further analysis.

**Figure 1:**
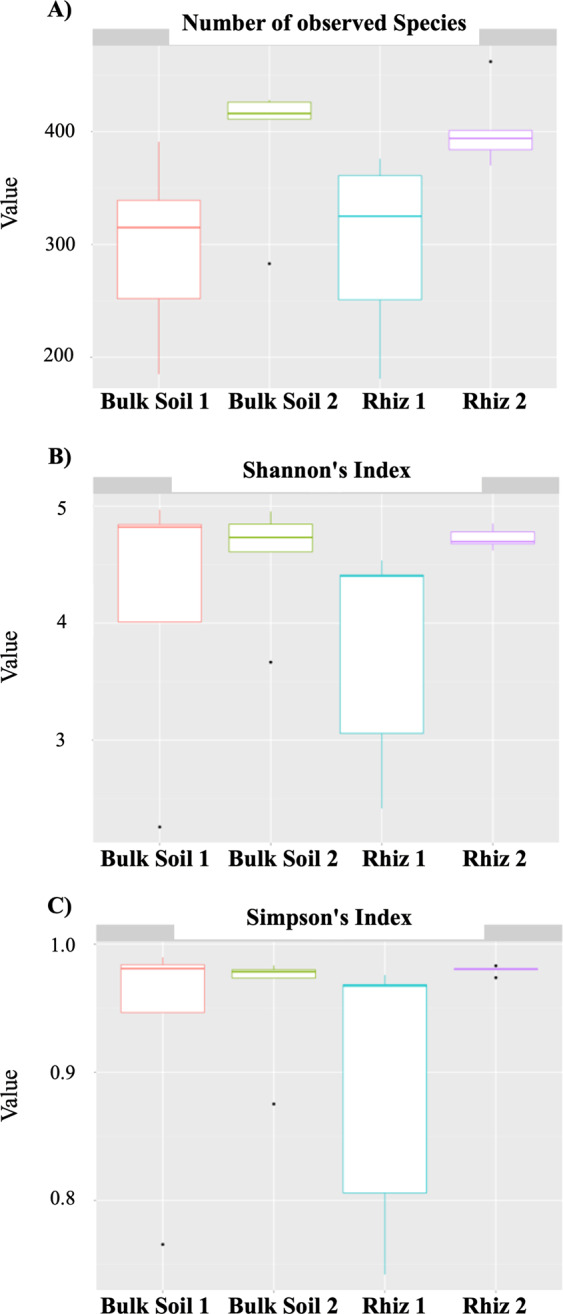
Alpha diversity evaluation: (**A**) Number of bacterial species detected in bulk soil and rhizosphere of *V. vinifera* at the two sampling times (**B**) Biodiversity (Shannon’s Index) of the microbial community found in bulk soil and the rhizosphere at the two sampling times (**C**) Simpson’s diversity index of the microbial community found in bulk soil and the rhizosphere at the two sampling times. Alpha diversity analysis was performed using R statistical software 3.5.1.

In order to measure alpha diversity (i.e., the local diversity of a community), the calculation of three estimators (number of bacterial species, Simpson’s Index and Shannon-Wiener’s Index) was performed. While the median number of bacterial taxa found in bulk soil and rhizosphere was comparable, it was higher during the second sampling (Bulk Soil 2, 416; Rhiz 2, 394) than at the first sampling (Bulk Soil 1, 315; Rhiz 1, 325) time (Fig. 1a). The median value of the Shannon-Wiener’s Index, that is an entropy measurement that increases according to the number of species in the sample, was highest in the bulk soil at the two sampling times and in the rhizosphere during the early fruiting stage (Fig. 1b). The Simpson’s Index, which is based on the probability of assigning two independent individuals, taken randomly from the community, into the same species, did not differ among the samples (Fig. 1c).

**Figure 2:**
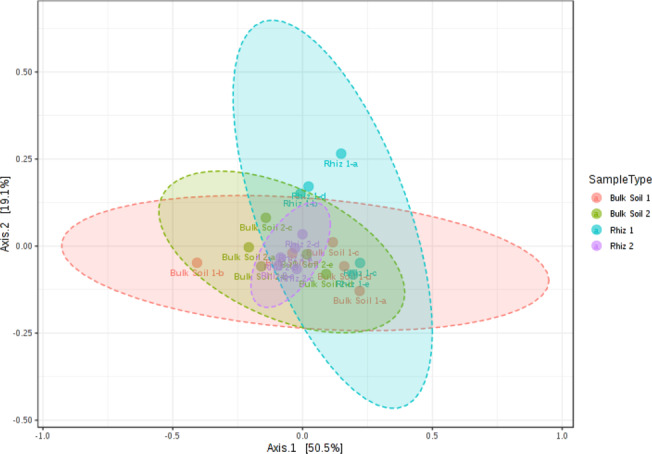
Beta diversity evaluation at the genus level: Principal Coordinate Analysis (PCoA) based on Bray–Curtis metrics shows the dissimilarity of microbial communities in bulk soil and rhizosphere according to sampling time. [ANOSIM] R: 0.259; p-value < 0.003. Beta diversity analysis was performed using MicrobiomeAnalyst, a free available on-line software (https://www.microbiomeanalyst.ca).

Beta diversity (the comparison of microbial communities based on their compositions) provides a measure of the distance or dissimilarity between each sample pair. Principal Coordinates Analysis (PCoA), performed on the recorded genera (Fig. 2), shows that the first axis explains 50.5% of the differences and the second one 19.1%. Shifts in microbial community according to the sampling time variable were more evident in rhizosphere samples than in bulk soil. However, the R (0.259) value obtained with ANOSIM indicates no significant dissimilarity among the considered groups.

### Description of the microbial communities

**Figure 3:**
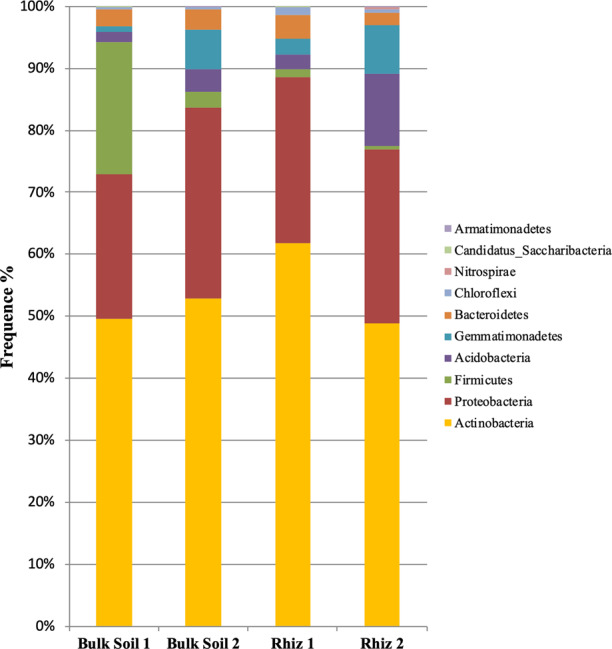
Microbial community composition in the bulk soil and rhizosphere of *V. vinifera* cv. Pinot Noir at the two sampling times (flowering and early fruiting stages) at the phylum level (top 8 taxa).

A total of 205,827 reads were used for phyla description. All the plots have been generated by considering the median value. Actinobacteria, followed by Proteobacteria and Firmicutes, were dominant in the bacterial communities both in bulk soil and in rhizosphere of the vineyard (Fig. 3). In particular, the abundance of Firmicutes was higher in bulk soil at first sampling compared to all the other samples.

Actinobacteria frequency did not change significantly in the samples (Bulk Soil 1 49.61%, Bulk Soil 2 52.80%, Rhiz 1 61.71% and Rhiz 2 48.76%). On the contrary, in bulk soil the frequency of readings ascribed to Proteobacteria and Firmicutes differed according to the sampling time: while the frequency of Proteobacteria and Firmicutes during the first sampling was 23.15% and 21.40%, respectively, during the second one it was 30.84% and 2.49%, (p = 0.032 and p = 0.008, respectively) (Fig. 3).

**Figure 4:**
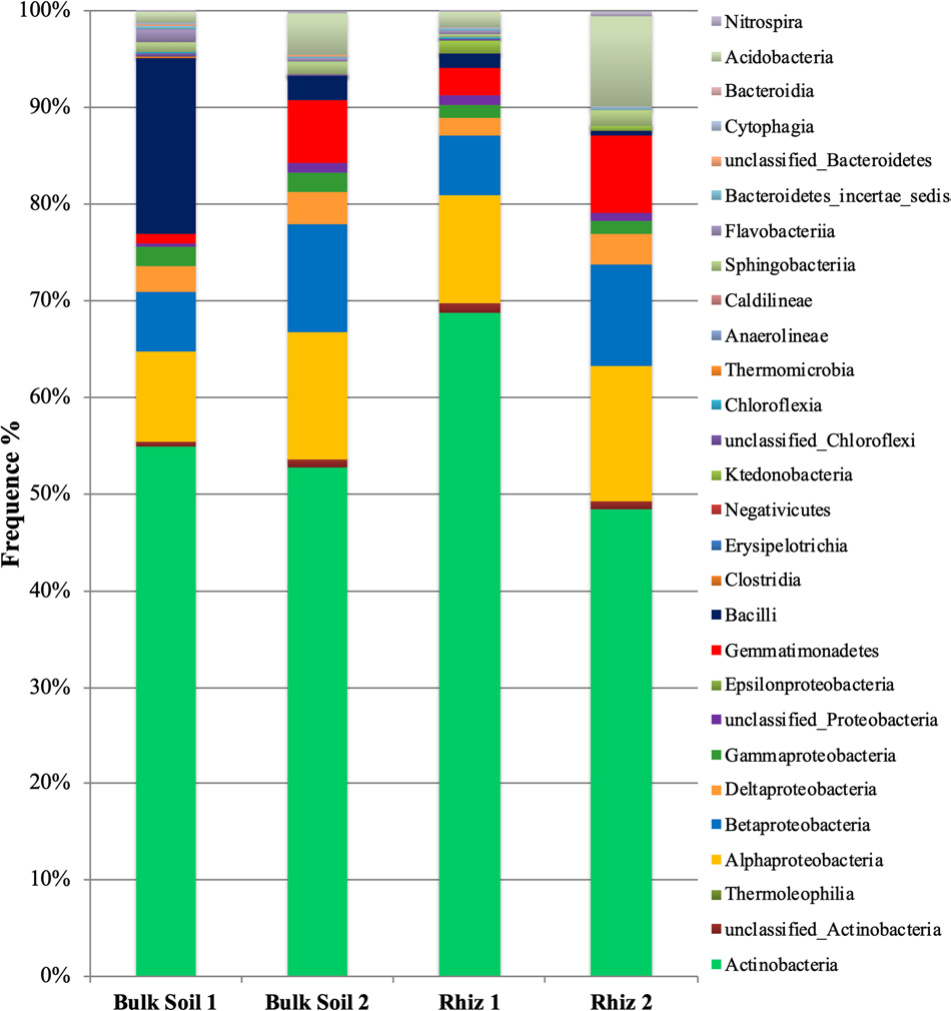
Microbial community composition in the bulk soil and rhizosphere of *V. vinifera* cv. Pinot Noir at the two sampling times (flowering and early fruiting stages) at the class level.

Similarly, the abundance of Acidobacteria and Gemmatimonadetes in the rhizosphere increased with time: while at flowering stage the frequency of readings corresponding to Acidobacteria and Gemmatimonadetes were 2.31% and 2.49%, respectively, at the early fruit development it was 11.63% (p = 0.032) and 7.99% (p = 0.008), respectively (Fig. 4).

Actinobacteria was the dominant class followed by α- and β-Proteobacteria (Fig. 4).

**Figure 5:**
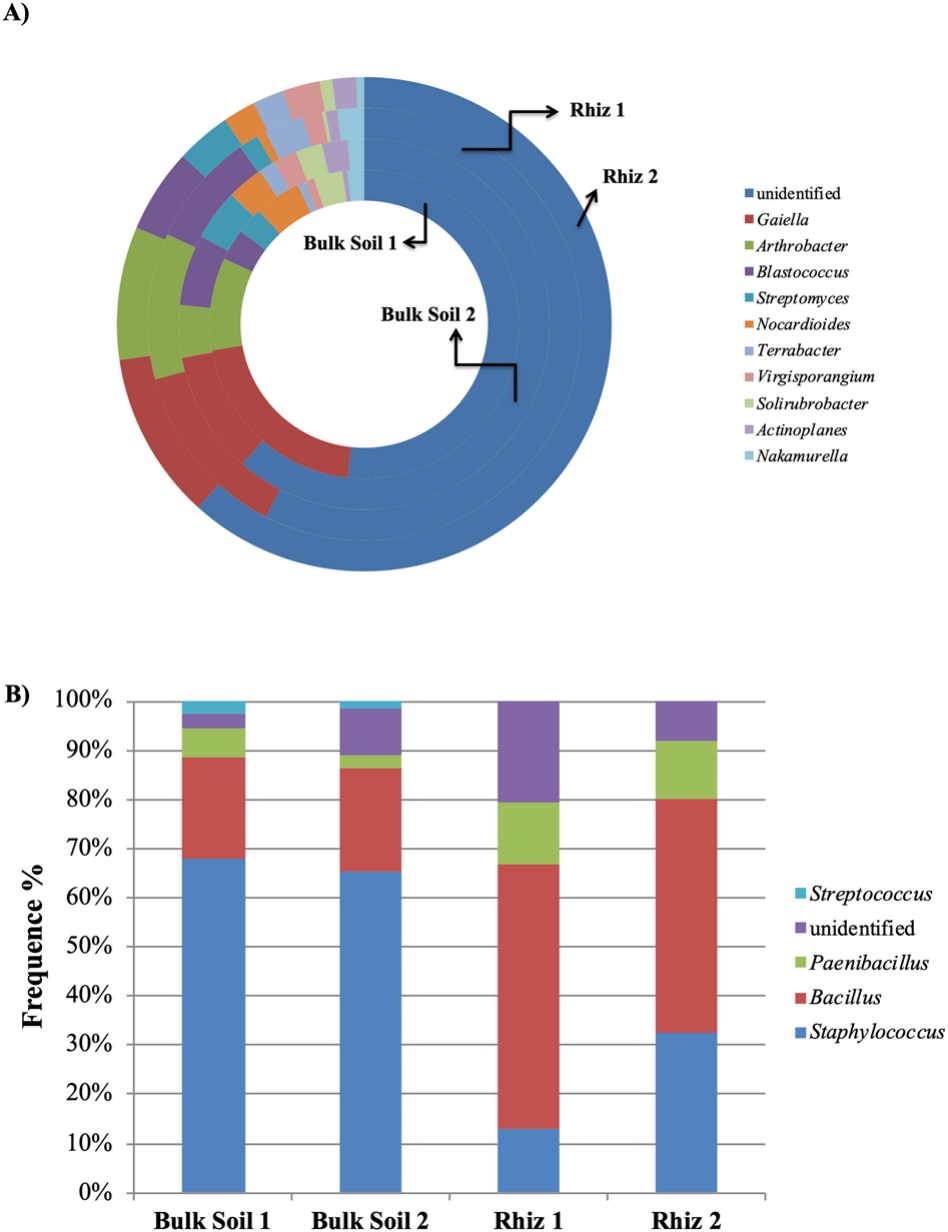
Distribution of the genera belonging to the class (**A**) Actinobacteria and (**B**) Bacilli, in bulk soil and rhizosphere of *V. vinifera* cv. Pinot Noir during the two sampling times (flowering and early fruiting stages). From the center to the edge Bulk Soil 1, Bulk Soil 2, Rhiz 1, Rhiz 2.

Actinobacteria, followed by unclassified_Actinobacteria, were the most represented classes belonging to the phylum Actinobacteria with high frequencies in all samples. Inside Actinobacteria, both in bulk soil and in the rhizosphere at the two sampling times, the dominant genera found were *Gaiella*, *Arthrobacter*, *Blastococcus, Streptomyces* and *Nocardioides* (Fig. 5A). The abundance of *Gaiella*, *Arthrobacter*, *Blastococcus* and *Streptomyces* did not change significantly and was similar in all soil samples. On the contrary, during the first sampling the frequency of *Nocardioides* was higher in bulk soil than in rhizosphere (Bulk Soil 1, 4.83% and Rhiz 1, 0.70%, p = 0.032) (Fig. 5A).

**Figure 6:**
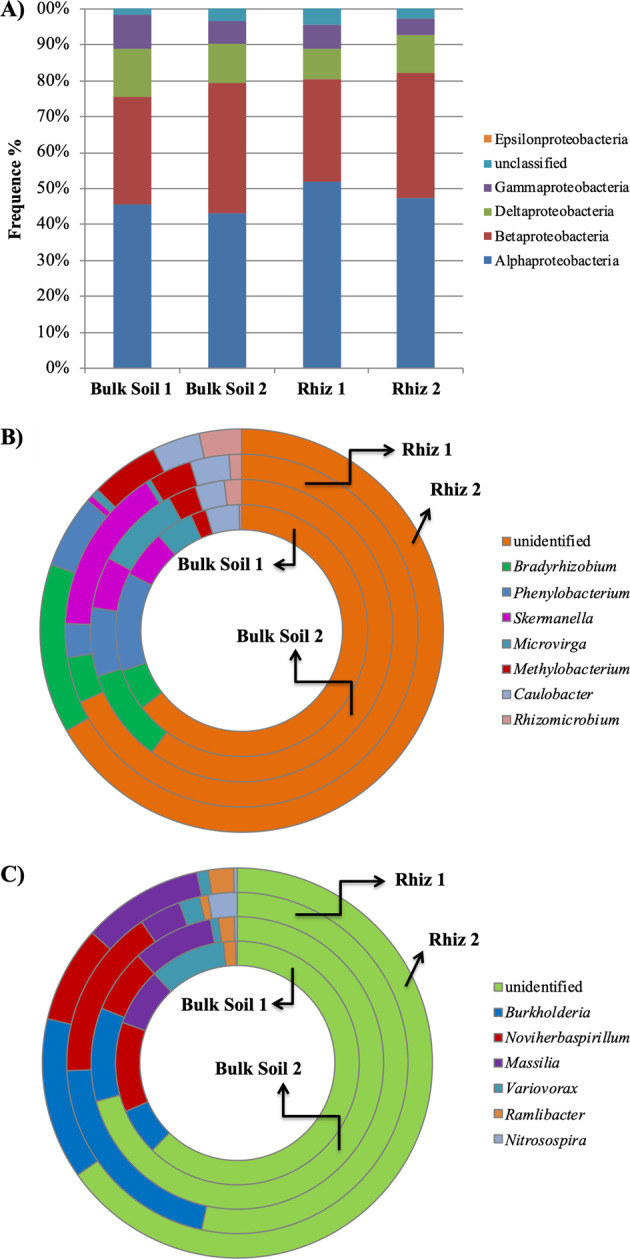
Distribution of (**A**) the different classes of Proteobacteria; (**B**) the genera belonging to the class α-Proteobacteria and (**C**) the genera belonging to the class β-Proteobacteria in bulk soil and rhizosphere of *V. vinifera* cv. Pinot Noir at the two sampling dates (flowering and early fruiting stages). From the centre to the edge Bulk Soil 1, Bulk Soil 2, Rhiz 1, Rhiz 2.

The distribution of the different Proteobacteria classes is reported in Fig. 6A. The results obtained by pyrosequencing indicated that α-, followed by β- and δ-Proteobacteria were dominant in all the soil samples.

The frequency of α-Proteobacteria in the bulk soil decreased with time (Bulk Soil 1 45.62% and Bulk Soil 2 43.16%, p = 0.008) (Fig. 6A). Similarly, the amount of reads ascribed to δ-Proteobacteria changed significantly according to the time variable both in rhizosphere (Rhiz1 8.30% and Rhiz2 10.60%, p = 0.016) and in bulk soil (Bulk Soil 1 13.11% and Bulk Soil 2 10.59%, p = 0.028).

Among the α-Proteobacteria, the most dominant identified genus was *Bradyrhizobium* followed by *Phenylobacterium*, *Skermanella* and *Microvirga*. According to the sampling time of the plant, the frequency of *Bradyrhizobium* differed in the rhizosphere (Rhiz 1, 4.19% and Rhiz 2, 13.57%; p = 0.016) and in bulk soil (Bulk Soil 1, 5.31% and Bulk Soil 2, 9.96%; p = 0.046) (Fig. 6B).

Significant frequency variations according to the sampling time occurred in the rhizosphere also for *Phenylobacterium* (Rhiz 1, 3.10% and Rhiz 2, 5.85%; p = 0.015). Moreover, the amount of readings corresponding to the genus *Microvirga* in bulk soil at the second sampling point was significantly higher than that recorded in the rhizosphere (Bulk Soil 2, 9.07% and Rhiz 2, 0.65%; p = 0.008). Instead, the frequency of *Skermanella* did not differ among the soil samples (Fig. 6B).

The predominant identified genera belonging to the β-Proteobacteria was *Burkholderia*, followed by *Noviherbaspirillum* and *Massilia*. The amount of readings ascribed to *Burkholderia* and *Noviherbaspirillum* did not change both in rhizosphere and in bulk soil at the two sampling times. In contrast, the abundance of *Massilia* in the rhizosphere changed significantly according to the sampling time, being higher at early fruit development than at the flowering time (Rhiz 1, 3.95% and Rhiz 2, 10.04%; p = 0.012) (Fig. 6C).

Inside the phylum Firmicutes, the amount of sequences corresponding to the class Bacilli in bulk soil changed with time (Bulk Soil 1 75.97% and Bulk Soil 2 13.76%, p = 0.008); in addition, their frequency during the first sampling time was much higher in bulk soil than in rhizosphere (Bulk Soil 1 75.97% and Rhiz 1 6.06%, p = 0.032). In the class Bacilli, the genus *Staphylococcus*, followed by *Bacillus* and *Paenibacillus* was dominant in all the soil samples. While the frequency of *Staphylococcus* and *Bacillus* genera did not changed significantly among the samples, the amount of readings ascribed to *Paenibacillus* in bulk soil was higher during the first sampling than in the second one (Bulk Soil 1 6.24% and Bulk Soil 2 2.54%, p = 0.047) (Fig. 5B).

In addition, besides the unclassified species, *Staphylococcus epidermidis* was predominant and their frequency did not differ among samples. The species *Staphylococcus haemolyticus* was detected only in bulk soil samples, although with low frequency (data not shown). The full list of the bacterial species found in the conventional pest managed vineyard with their frequencies are reported in supplementary materials (Table S1).

Finally, based on the genera recorded in the samples, the heatmap visualization (Fig. S2, supplementary material) clearly indicated a huge variability inside the same sample. However, the Pattern Search analysis, showed that the genera more affected by sampling time were *Pseudomonas, Nakamurella, Bacillus*, and *Acidobacteria* GP7 (Fig. S3, supplementary material).

## Discussion

The effect of the plant species, cultivar and age on the microbial community structure have been described in different studies^28–32^; similarly, the impact of soil management, such as use of herbicides and pesticides, both on soil and rhizosphere microbial communities have been characterized in a number of papers^33–35^. Taking into account the rhizodeposition, the amount and the quality of root exudates changes according to the phenological stage of the plant; these changes can exert an effect on the microbiota associated with the host plant^36,37^.

In a previous work, we analyzed the biodiversity of the bacterial rhizosphere bacteriome of grapevine (cv. Pinot Noir) in an integrated pest managed vineyard. The main result of this paper was the demonstration that the bacterial community associated with grapevine differed from that of the bulk soil and these variations were independent of the phenological stage of the plant. While this metabarcoding analysis provided a description of the whole bacterial community, a metaproteome approach was further applied to this environment in order to study the active species of this bacteriome and allowed us to understand their function^26^.

Conventional viticulture can induce several negative effects on soil microbial communities, mainly due to fungicide/pesticide treatments^20,24^, by acidification of the soil due to fertilizer input^38,39^, and by tillage practices^40^. These agricultural practices can thus modulate plant-microbe interactions.

In this work, we assessed if the bacterial fraction of the microbiota associated to grapevine (cv. Pinot Noir) cultivated in a conventional pest managed vineyard responds to the rhizosphere effect (plant presence, comparison between bulk soil vs. rhizosphere) or to the sampling time (comparison between first sampling, flowering and second sampling, early fruit development).

The number of species detected at the second sampling was higher than that at the first sampling both in bulk soil and in rhizosphere. Similarly, microbial biodiversity, measured as Shannon’s Index was higher during the second sampling than at the first one mainly in the rhizosphere. Overall, our results suggest that the shifts in the bacterial communities occurred according to sampling time. However, such variations of the microbial community composition were detected both in the rhizosphere and in the bulk soil, especially in terms of the number of taxa. Therefore, it’s possible to hypothesize that the phenological stage of the plant is not the only factor modulating the bacteriome composition, but the pressure induced by the chemical treatments (scheduled by Italian law in this kind of management) must be considered, as well. PCoA indicated the occurrence of time-related shifts in the microbial community composition, more evident in the rhizosphere samples, than in the bulk soil. Differences, however, were not significant due to the high variability of the bacteriome composition among the five subsamples (see Fig. S2, supplementary material). Indeed, it is well known that soil is a very heterogeneous and complex system in which microorganisms and other soil components are irregularly distributed^41^. Also in uniformly managed systems, which are considered more homogenous than natural ones, biological processes (e.g., growth and colony formation) may induce the formation of microbial aggregates at various spatial scales^42^. Therefore, soil microbiota is characterized by patchy distributions at a scale ranging from several millimeters to several meters^43,44^.

Regarding phyla distribution, our data indicated that the predominant phyla, in all the soil samples, were Actinobacteria (with high frequencies > 50%), Proteobacteria, Firmicutes and Acidobacteria. These results are in partial agreement with other recent works focused on the structure of microbial communities in the vineyard ecosystem^25,45–48^. Proteobacteria, Actinobacteria, Firmicutes, Bacteroidetes and Acidobacteria were the most represented phyla in a conventional pest managed vineyard, found through DGGE methods^48^.

Members of Actinobacteria and Proteobacteria are known to be the dominant phyla in soil, and are supposed to be involved in the degradation of organic matter^49^, the production of secondary metabolites^50^, P solubilization and N_2_ fixation, thus playing an essential role in nutrient cycling^51^, in the enhancement of soil fertility and crop productivity^52^, and in plant growth promotion^53,54^. These physiological traits could be considered at the base of the biostimulant activity of the strains belonging to these phyla^26^.

As already reported in Novello *et al*.^25^, describing the rhizosphere bacteriome of a vineyard subjected to IPM, *Gaiella*, a non-motile rod-shaped that stains Gram-negative, was the dominant genus of Actinobacteria in all the samples. Bacteria belonging to this genus are strictly aerobic, oxidase and catalase positive, and the type species is *Gaiella occulta*, described for the first time in 2011 by Albuquerque and colleagues^55^.

Among the Proteobacteria, the most represented classes found in the vineyard (at both sampling times) were Alpha, Beta and Gamma-Proteobacteria. In general, the amount of sequences ascribed to all Proteobacteria classes was higher, both in soil and rhizosphere, at the second sampling (early fruiting stage) than at the first sampling (flowering stage). Several studies have shown that rhizospheric fungal and bacterial communities of a wide range of plants (i.e., *Arabidopsis* sp., *Medicago* sp., maize, pea, wheat and sugar beet) change according to a plant developmental stage^28,31,56–58^. It has been hypothesized that the increase of Gamma-Proteobacteria in the rhizosphere at different times can be ascribed to a higher or more favorable organic matter release during plant growth^59^. Moreover, in recent years, the effect of herbicides and pesticides on bacterial communities in the rhizosphere of corn and soybean demonstrated that all Proteobacteria classes, and especially Gamma-Proteobacteria, increased following herbicide treatment (glyphosate)^60^. On the contrary, in wheat rhizosphere the dominance of Proteobacteria decrease with plant age^61^.

Regarding Alpha-Proteobacteria the most represented identified genus was *Bradyrhizobium* that is known for its ability to promote plant growth and fix nitrogen^62^, while *Burkholderia* was the predominant genus of the Beta-Proteobacteria. The genus *Burkholderia* is represented by an interesting and complex bacterial taxonomic unit including a variety of species inhabiting different ecological niches such as soil, plant rhizosphere, water and humans^63^. In recent years, a growing number of *Burkholderia* strains and species have been reported as plant-associated bacteria with different intimacy degree with the plant, ranging from free-living to epiphytic and endophytic. While several strains are known to behave as biocontrol agents, to improve nitrogen fixation and enhance plant tolerance to environmental stresses, some species are phytopathogens. On the other side, some species/isolates can be opportunistic or obligate (*B. mallei* and *B. pseudomallei*) pathogens causing human diseases^64^.

Among Firmicutes, *Staphylococcus* was dominant in bulk soil and *Bacillus* in rhizosphere. The high frequency of sequences ascribed to the genus *Bacillus* had already been observed in an integrated pest managed vineyard, located close to this conventional managed vineyard, by Novello *et al*.^25^. On the contrary, sequences corresponding to the genus *Staphylococcus*, that were absent in the integrated pest managed vineyard previously described^25^, have been found especially in bulk soil and, to a lower extent, in the vine rhizosphere. In particular, *Staphylococcus epidermidis* was the dominant species while *Staphylococcus haemolyticus* was present in soil samples although with low frequency. The occurrence of *S. haemolyticus* from internal tissue of plants has been previously documented^65^. Both these bacterial species are classified as human opportunistic pathogens^66,67^. Some opportunistic human bacterial pathogens are even able to colonize plant tissues^68^ and the occurrence of these bacteria in the rhizosphere and soils received much attention in the last years. In fact, different works reported the presence of possible opportunistic human pathogenic bacteria associated with plant roots of several species such as potato, strawberry and rice^69–74^.

Yousaf and collaborators^75^, by pyrosequencing approach, found opportunistic human pathogens in the grapevine endosphere; in this work, four bacterial genera (*Burkholderia*, *Propionibacterium*, *Staphylococcus* and *Clostridium*), which include opportunistic human pathogens, were detected. In the same year, Campisano and colleagues reported the presence of opportunistic human pathogens in grapevine^76^. Surprisingly, no sequences belonging to human pathogens have been find in our previous work performed on the bacteriome of a vineyard subjected to integrated pest management^25^. In an attempt of comparison between the bacteriome described in this paper and that reported in Novello *et al*.^25^, the main result is the variation of the abundance of Acidobacteria group, that was higher in the vineyard subjected to conventional management than in the IPM vineyard. This could be ascribed both to the pH of the soil (more acidic in the conventional vineyard than in IPM) and to the acidification induced by pesticide treatment. In contrast, the frequency of Actinobacteria and Proteobacteria was similar in the two vineyards. Moreover, the occurrence of members of the genus *Bacillus* was higher in the conventionally managed vineyard than in IPM. We think that, according with this data, *Bacillus* and Acidobacteria can be considered as a possible marker of stressed soil in agreement with Berlanas and coworkers^12^. However, the differences between the soil chemical-physical characteristics of the two vineyards hampers a realistic comparison.

Further researches are needed to: i) describe the “actors” and “roles” of the microbial community members by applying the metagenome/metaproteome approach described by Bona *et al*.^26^ to this conventional managed vineyard; ii) found new bacterial strains with plant beneficial physiological traits to be used as biostimulants in degraded vineyards; iii) complete the microbiome description characterizing arbuscular mycorrhizal fungal communities. Following this route, new perspectives in the vineyard ecosystem knowledge and management will be opened, with a positive impact on the winemaking procedure as well as on the environmental and consumer health.

In conclusion, this study, together with the other papers published by our group^25,26^, is one of the missing piece to enhance the understanding of the microbiota of Pinot Noir grapevine in the perspective of more sustainable viticulture at global scale.

## Materials and Methods

### Soil sampling

**Figure 7:**
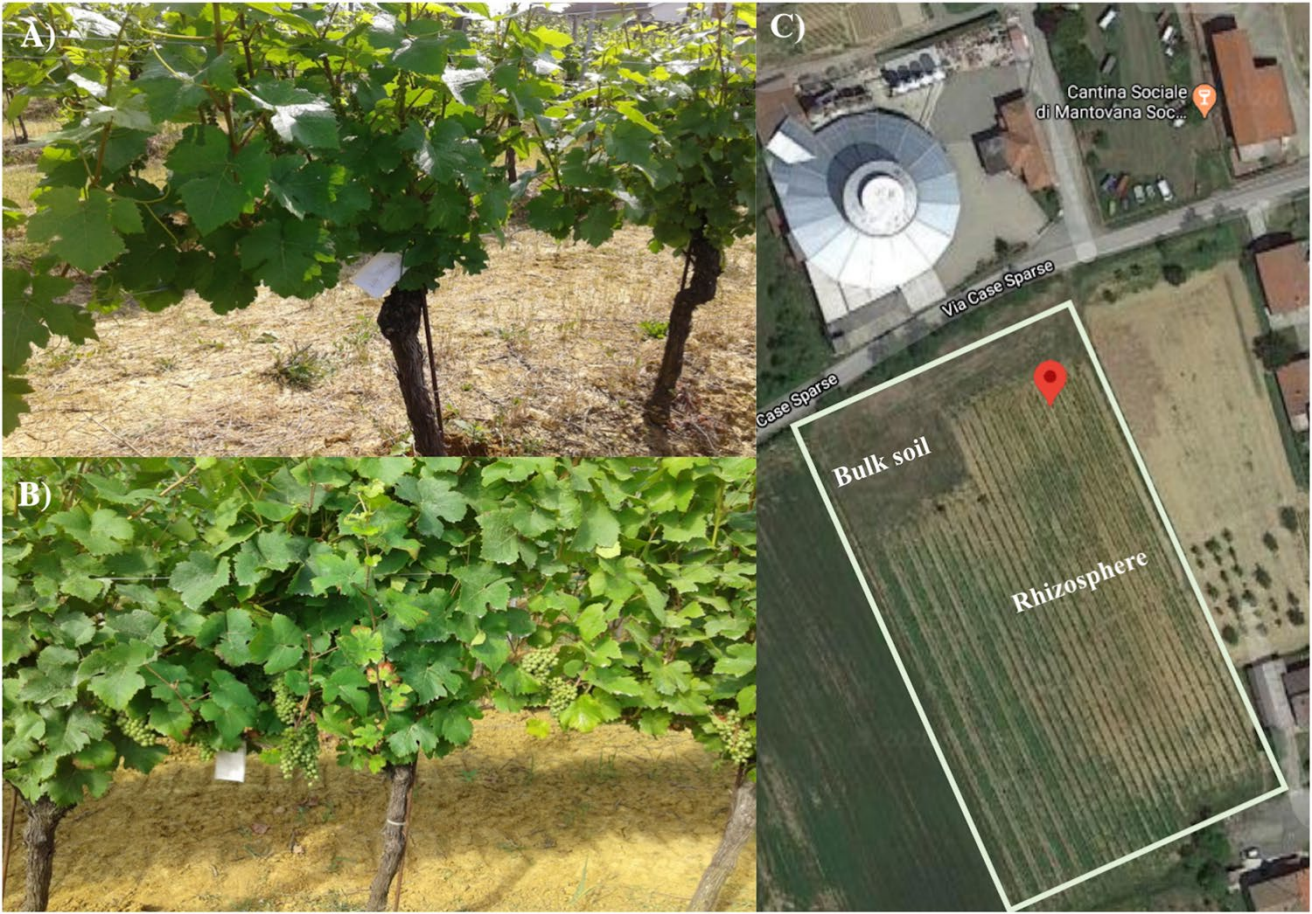
Grapevine at the two sampling times corresponding to two phenological stages: (**A**) flowering and (**B**) early fruit development. (**C**) Map of the sampling site: bulk soil and rhizosphere are indicated in the map; the red tag indicates the coordinates specified in the materials and methods. The map image was produced by the authors using Google Maps (https://www.google.com/maps/@44.730294,8.6226556,681m/data=!3m1!1e3).

The conventional pest managed vineyard is located in Mantovana (Predosa, Alessandria, Italy): Latitude: 44.730294°N, Longitude: 8.6226556 °E and Altitude: 215.35 m a.s.l. (Fig. 7C). The soil was clay loam (Sand 45.0%, Silt 26.8%, Clay 28.2%), acid (pH 5.99), with a total organic carbon 6.4 g/Kg, total Nitrogen 0.70 g/Kg, C/N ratio 9.06 and CEC 15.8 meq/Kg. Data regarding temperature, humidity and rainfall are reported in Novello *et al*.^25^.

Soil samplings were performed at two times corresponding to different phenological stages of *Vitis vinifera* cv. Pinot Noir (Rootstock SO4). The first sampling was carried out in May 2014, corresponding to the flowering time and second sampling in July 2014 during the early fruit development (Fig. 7A,B).

The conventional management of the “Cantina Mantovana” vineyard was based on treatment with different chemicals: the herbicide glyphosate between the vineyard lines (in June), fungicide against *Oidium* spp. (Trifloxistrobin), fungicide against *Peronospora* spp. (Fosetyl-Al + copper) in June and July, one insecticide (Thiamethoxan) in July and, finally two sulphur treatments.

The bulk soil (Bulk Soil 1 and Bulk Soil 2, for each sampling date) and the soil associated with the roots of *Vitis vinifera* cv. Pinot Noir (Rhiz 1 and Rhiz 2, for each sampling date), five samples per each kind, were collected at a depth of 30 cm (topsoil), after removing the surface layer (3.0–5.0 cm). Three soil cores were taken in the proximity of the stem, therefore a total of fifteen cores were taken for each kind of soil. The roots entrapped in the soil cores collected 3 cm close to the stem were considered for the sampling of rhizosphere soil. The soil adhering to these roots was removed using sterile gloves. As recommended by the Italian law (GU 179/2002), for soil microbiological characterization analysis, the three subsamples of rhizosphere and bulk soils were then pooled in order to obtain a homogeneous sample^25^. Therefore, for each sample and each sampling time, five biological replicates have been sequenced. Soil samples were then stored at −20 °C for further DNA extraction.

### DNA extraction

Metagenomic DNA was directly extracted from 0.25 g of soil by using the Power Soil® DNA Isolation Kit (MO BIO Laboratories, Inc., Carlsbad, CA) according to the manufacturer’s instructions. The DNA was firstly visualized in electrophoresis on an agarose gel 0.8% in TAE 1X buffer and then precipitated with ethanol. The amount and the purity of DNA were measured by spectrophotometric absorbance at λ 260 nm and at λ 230/280 nm, before and after the precipitation step, respectively^25^.

### DNA amplification and Roche 454 pyrosequencing

Amplification of DNA extracted from the five samples of both bulk soil (Bulk Soil) and rhizosphere (Rhiz), collected during flowering and early fruit development, was performed with the primer pairs for V1 (5'- AGAGTTTGATCCTGGCTCAG–3')^77^ and V4 (5'- CTACCAGGGTATCTAATC-3')^78^ regions of 16 S rDNA, tagged with Multiplex Identifier sequences for 454 Pyrosequencing (Roche). The PCR reaction was performed in a Techne thermocycler (TC512, Bibby Scientific, Riozzo di Cerro al Lambro, Italy). Amplification conditions were: initial denaturation at 94 °C for 5 min; 34 cycles at 94 °C for 1 min, 60 °C for 1 min, and 72 °C for 5 min; and a final elongation at 72 °C for 10 min. Five ng of amplified DNA were contained in each reaction mixture (20 μl) together with 100 μM of dNTPs DNA, 1.5 mM MgCl_2_, 1× Buffer [67 mM Tris–HCl pH 8.8; 16.6 mM (NH_4_)_2_SO_4_; 0.01% Tween-20; MgCl_2_ 5 mM], 0.08 U of Taq DNA Polymerase (Thermofisher) and DMSO 5%.

PCR products were used for pyrosequencing with 454 technology; amplicons were amplified in droplet water in oil emulsions. DNA-carrying beads were loaded into individual wells on a PicoTiter (Roche Diagnostics S.p.A.) plate and surrounded by enzyme beads (sulfurylase luciferase). Nucleotides were flowed one at a time over the plate; after the template-dependent incorporation, pyrophosphate was released and converted to light through luciferin/luciferase enzymatic reaction. The light signals were represented in flowgrams and analysed; a nucleotide sequence was determined for each read with the GS Amplicon Variant Analyzer software^25^.

### Bioinformatic and statistical analyses

Data were analyzed using a custom bioinformatics pipeline (SmartSeq s.r.l.). Raw sequence reads were demultiplexed to obtain a single file for sample. During this process, reads that met the following criteria were discarded: (1) read length <than 200 nt, (2) average Phred quality score^79^ <than 25, (3) reads containing at least one ambiguous base.

For each sample, the taxonomy assignment up to genus level was performed using RDP (https://rdp.cme.msu.edu) classifier^80^ and species-level resolution was attained by blasting reads against a core set of the RDP database.

Sequences were clustered according to similarity thresholds (≥97%) and the representative sequence of each cluster was identified with the name of the corresponding RDP hit for all taxonomic levels.

Finally, a table with absolute abundance for all soil samples was used as input for the analysis with RAM package of R statistical software 3.5.1, released in 2018 (https://www.r-project.org/), to obtain the alpha diversity graphs and the biodiversity indices (Shannon-Wiener’s index, Simpson’s index, number of observed species)^81^.

Statistical analysis was performed with R statistical software^81^. Data were compared by non-parametric Mann-Whitney U test with cut-off significance at P < 0.05 to assess differences between treatments^81^.

Principal Coordinates Analysis (PCoA) has been obtained by using R Phyloseq version 1.19, and calculated with Bray-Curtis dissimilarity Index. The heatmap visualization and the Pattern Search graph have been obtained with the online tool “MicrobiomeAnalyst”, a free available on-line software (https://www.microbiomeanalyst.ca)^82^.

## Supplementary information

Supplementary Information.

## Data Availability

The genomic datasets generated and/or analyzed during the current study are available in NCBI using BioProject ID: PRJNA600377 containing the following BioSamples: SAMN13818351, SAMN13818352, SAMN13818353, SAMN13818354. Project Name: Discovering the microbiota of *Vitis vinifera* cv. Pinot Noir in a conventionally managed vineyard.
