# Author Correction: Discovering the bacteriome of *Vitis vinifera* cv. Pinot Noir in a conventionally managed vineyard

**DOI:** 10.1038/s41598-021-88232-5

**Published:** 2021-04-19

**Authors:** Elisa Gamalero, Elisa Bona, Giorgia Novello, Lara Boatti, Flavio Mignone, Nadia Massa, Patrizia Cesaro, Graziella Berta, Guido Lingua

**Affiliations:** 1 Università del Piemonte Orientale, Dipartimento di Scienze e Innovazione Tecnologica, Viale T. Michel 11, 15121 Alessandria, Italy; 2 Università del Piemonte Orientale, Dipartimento di Scienze e Innovazione Tecnologica, Piazza San Eusebio 5, 13100 Vercelli, Italy; 3 SmartSeq s.r.l., spin-off of the Università del Piemonte Orientale, Viale T. Michel 11, 15121 Alessandria, Italy

Correction to: *Scientific Reports* https://doi.org/10.1038/s41598-020-63154-w, published online 15 April 2020

The original version of this Article contained an error in the order of the Figures. Figures 1, 2, 3, 4, 5, 6 and 7 were incorrectly published as 2, 3, 4, 5, 6, 7 and 1. The Figure legends were correct.

The original Figures 1, 2, 3, 4, 5, 6, 7 and accompanying legends appear below.

**Figure 1:**
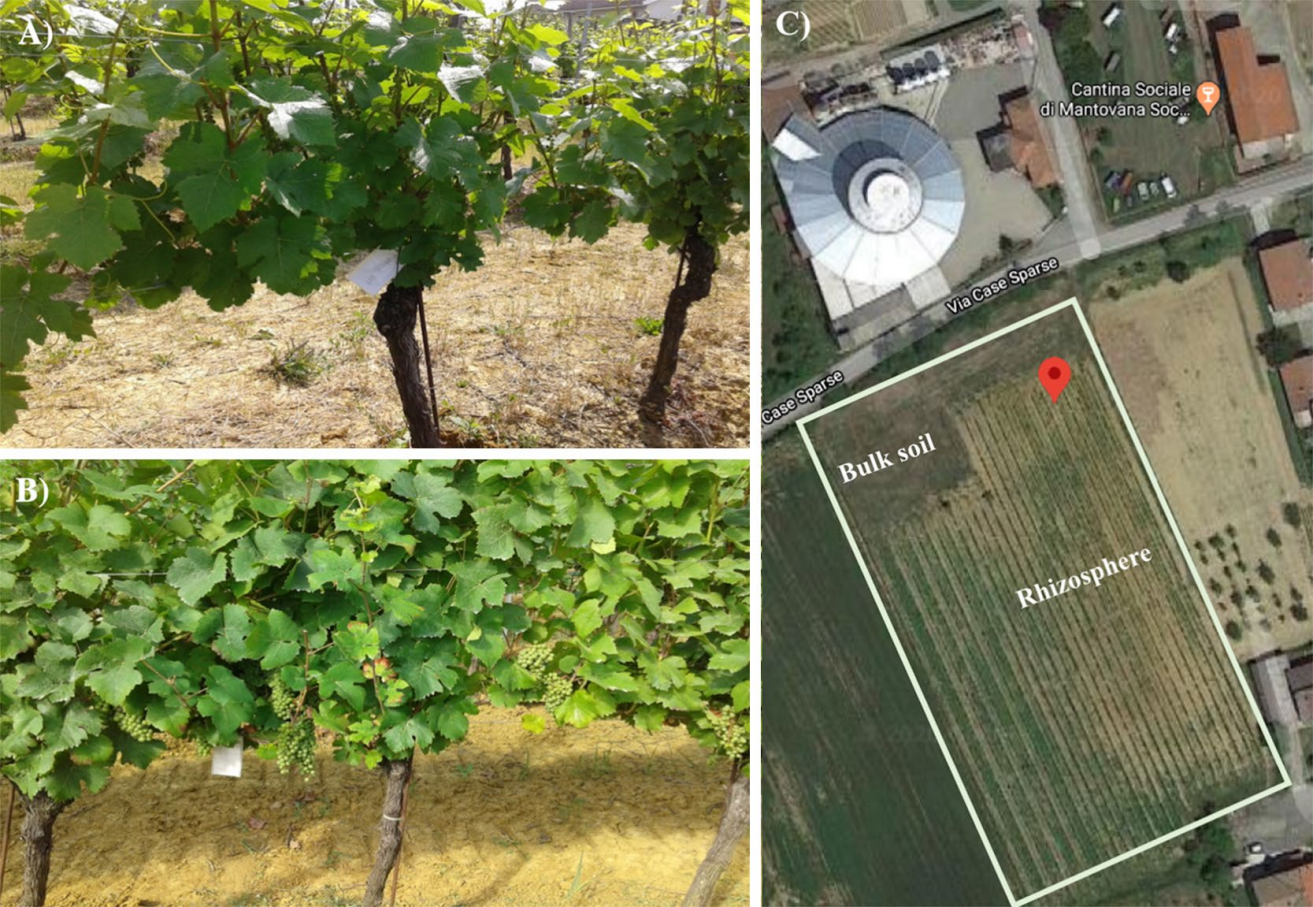
Grapevine at the two sampling times corresponding to two phenological stages: (**A**) flowering and (**B**) early fruit development. (**C**) Map of the sampling site: bulk soil and rhizosphere are indicated in the map; the red tag indicates the coordinates specified in the materials and methods. The map image was produced by the authors using Google Maps (https://www.google.com/maps/@44.730294,8.6226556,681m/data=!3m1!1e3).

**Figure 2:**
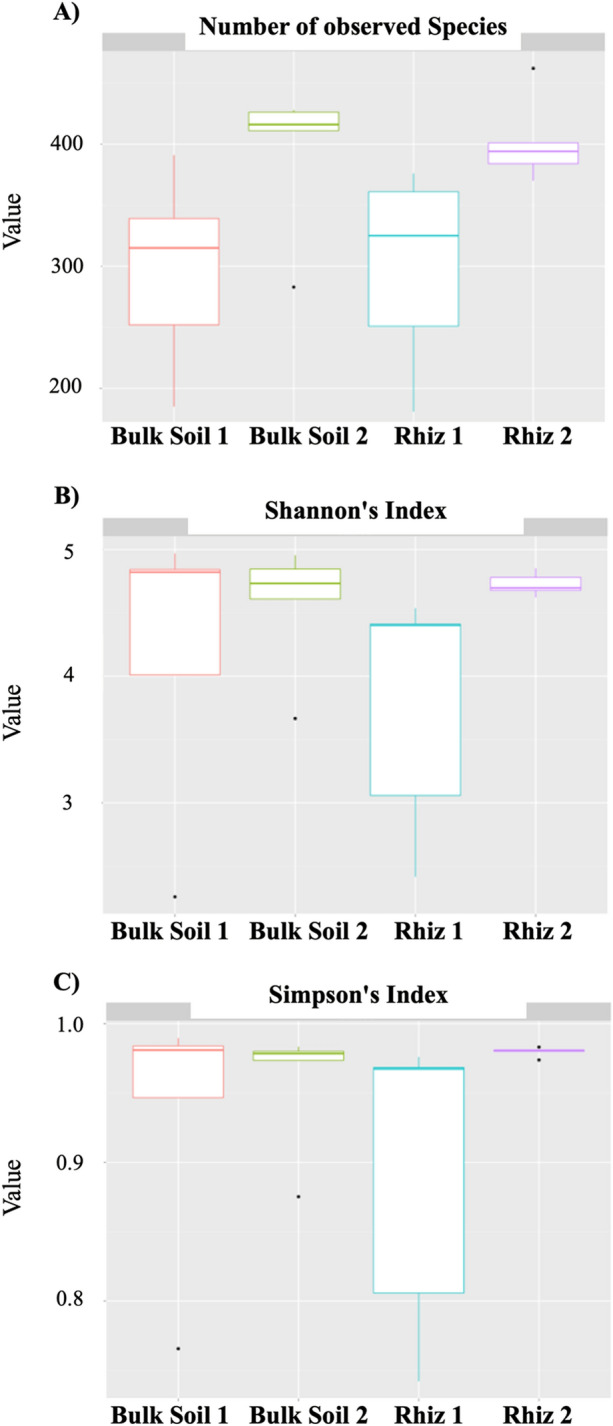
Alpha diversity evaluation: (**A**) Number of bacterial species detected in bulk soil and rhizosphere of *V. vinifera* at the two sampling times (**B**) Biodiversity (Shannon’s Index) of the microbial community found in bulk soil and the rhizosphere at the two sampling times (**C**) Simpson’s diversity index of the microbial community found in bulk soil and the rhizosphere at the two sampling times. Alpha diversity analysis was performed using R statistical software 3.5.1.

**Figure 3:**
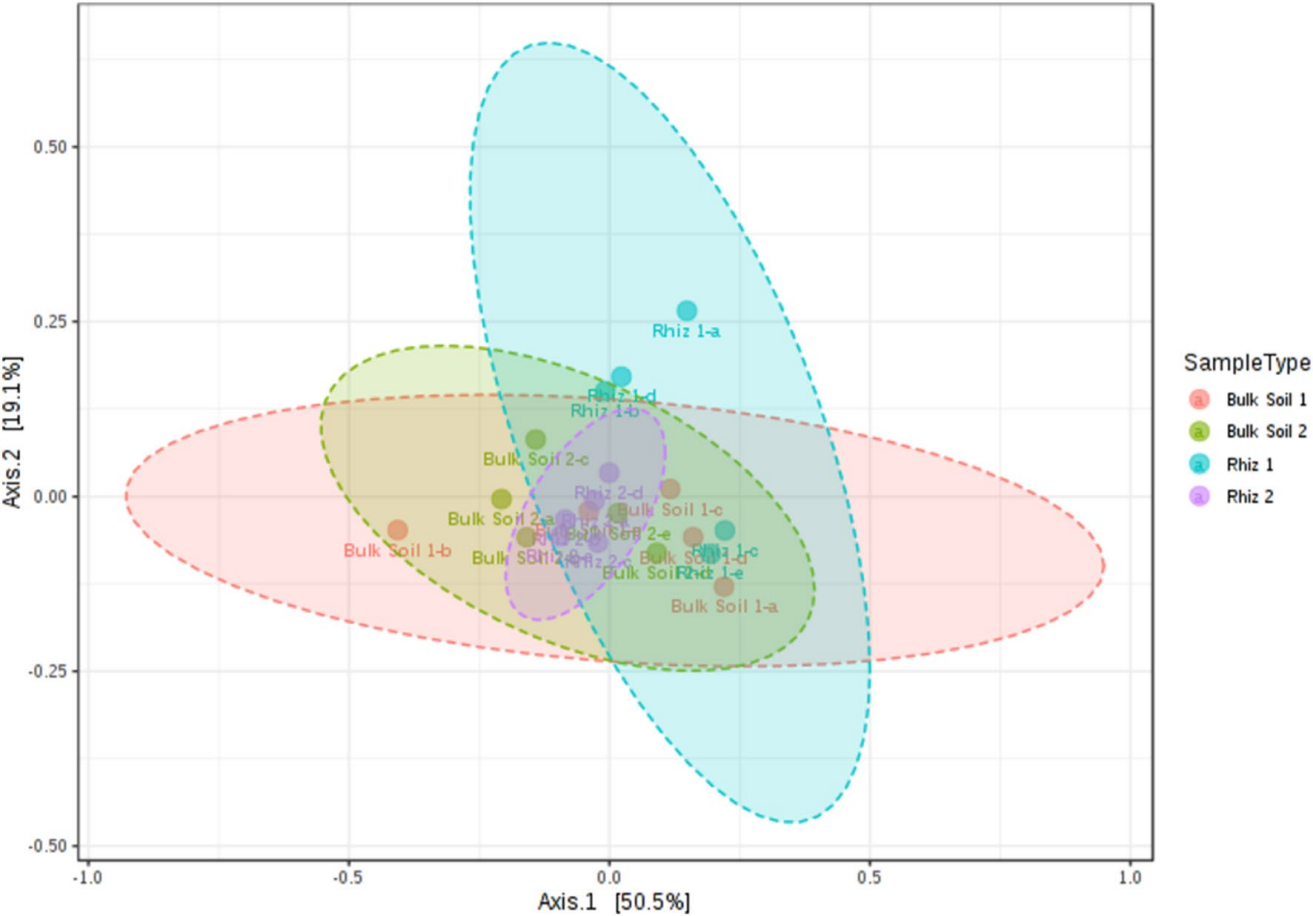
Beta diversity evaluation at the genus level: Principal Coordinate Analysis (PCoA) based on Bray–Curtis metrics shows the dissimilarity of microbial communities in bulk soil and rhizosphere according to sampling time. [ANOSIM] R: 0.259; p-value < 0.003. Beta diversity analysis was performed using MicrobiomeAnalyst, a free available on-line software (https://www.microbiomeanalyst.ca).

**Figure 4:**
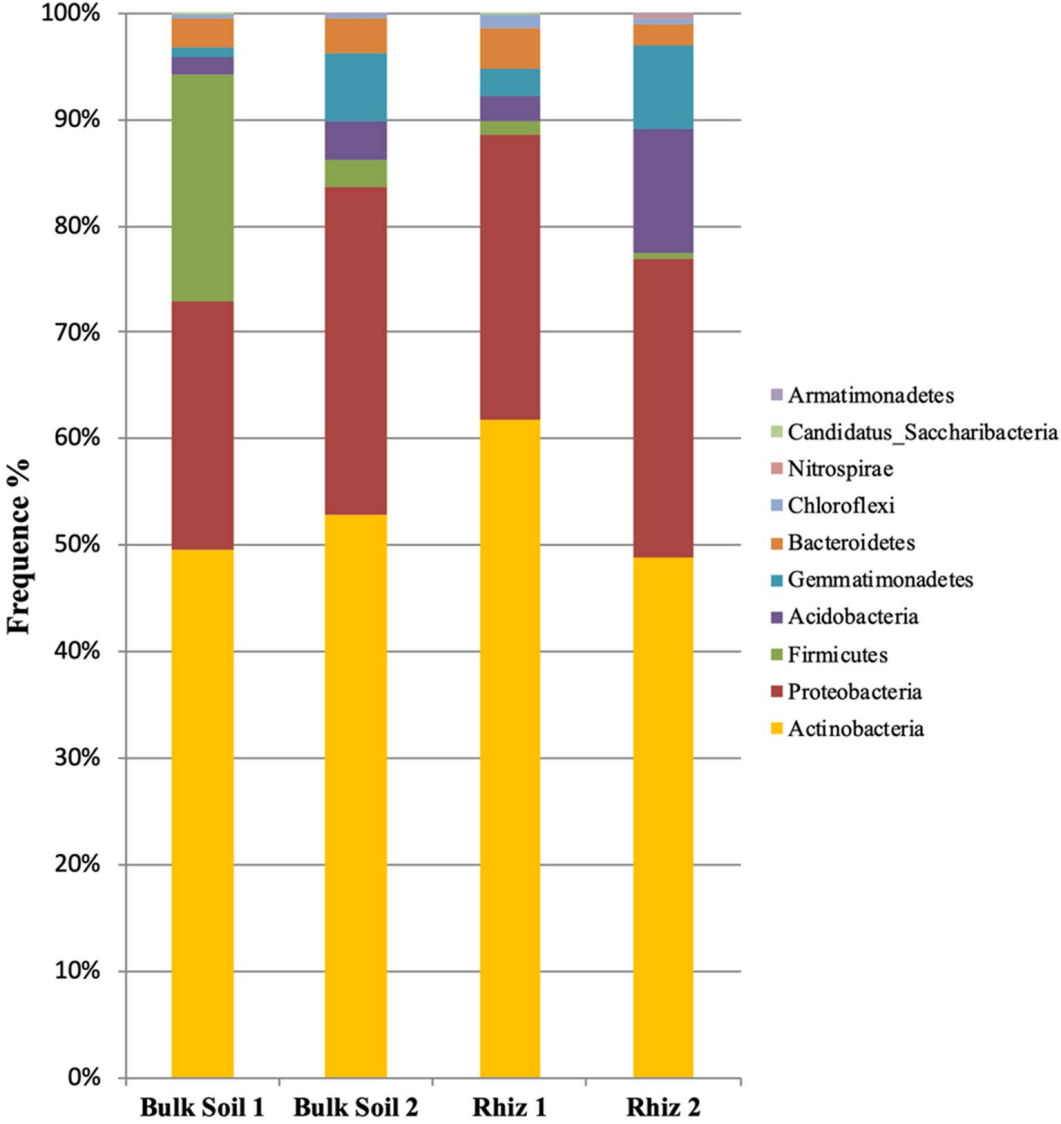
Microbial community composition in the bulk soil and rhizosphere of *V. vinifera* cv. Pinot Noir at the two sampling times (flowering and early fruiting stages) at the phylum level (top 8 taxa).

**Figure 5:**
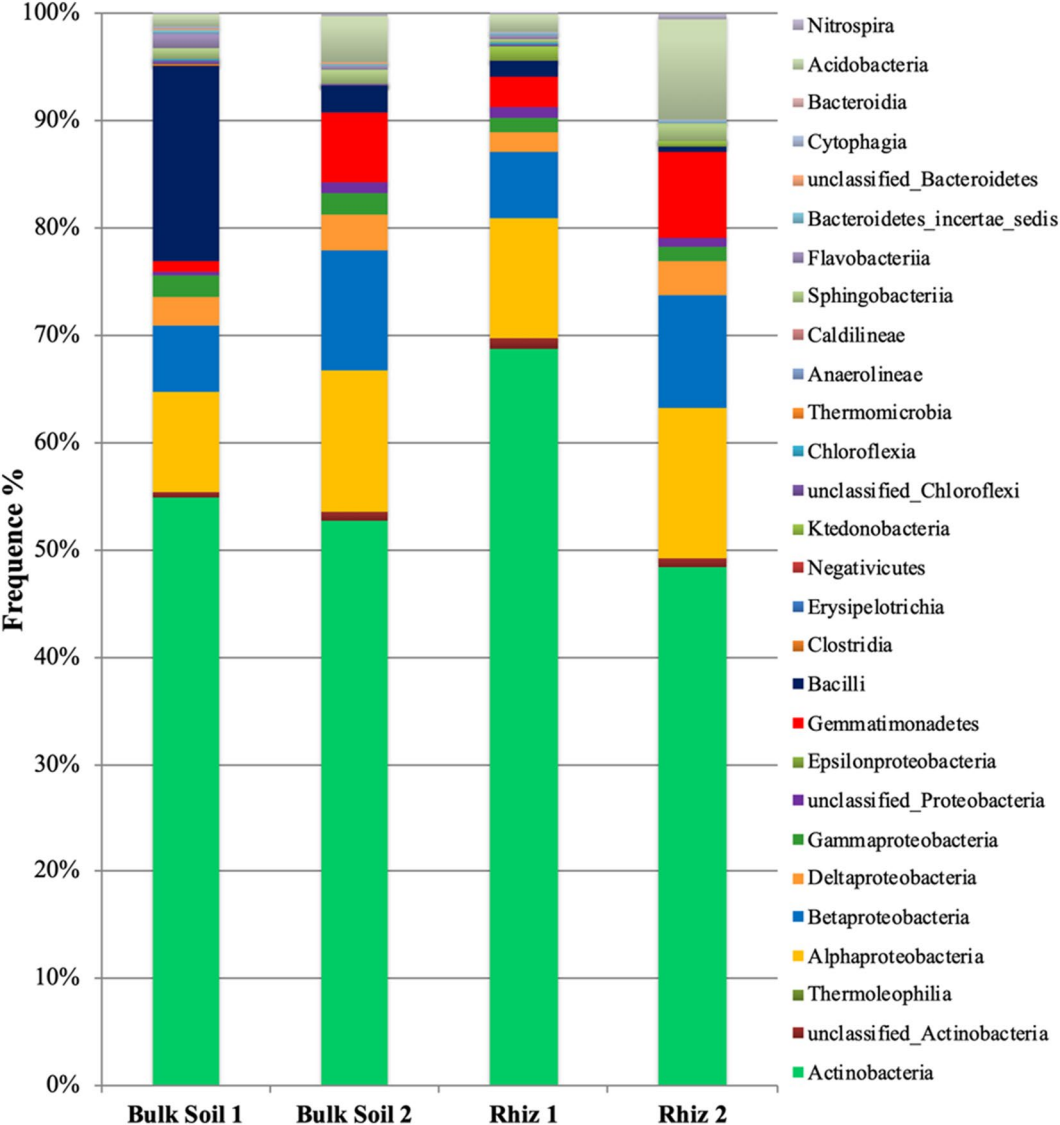
Microbial community composition in the bulk soil and rhizosphere of *V. vinifera* cv. Pinot Noir at the two sampling times (flowering and early fruiting stages) at the class level.

**Figure 6:**
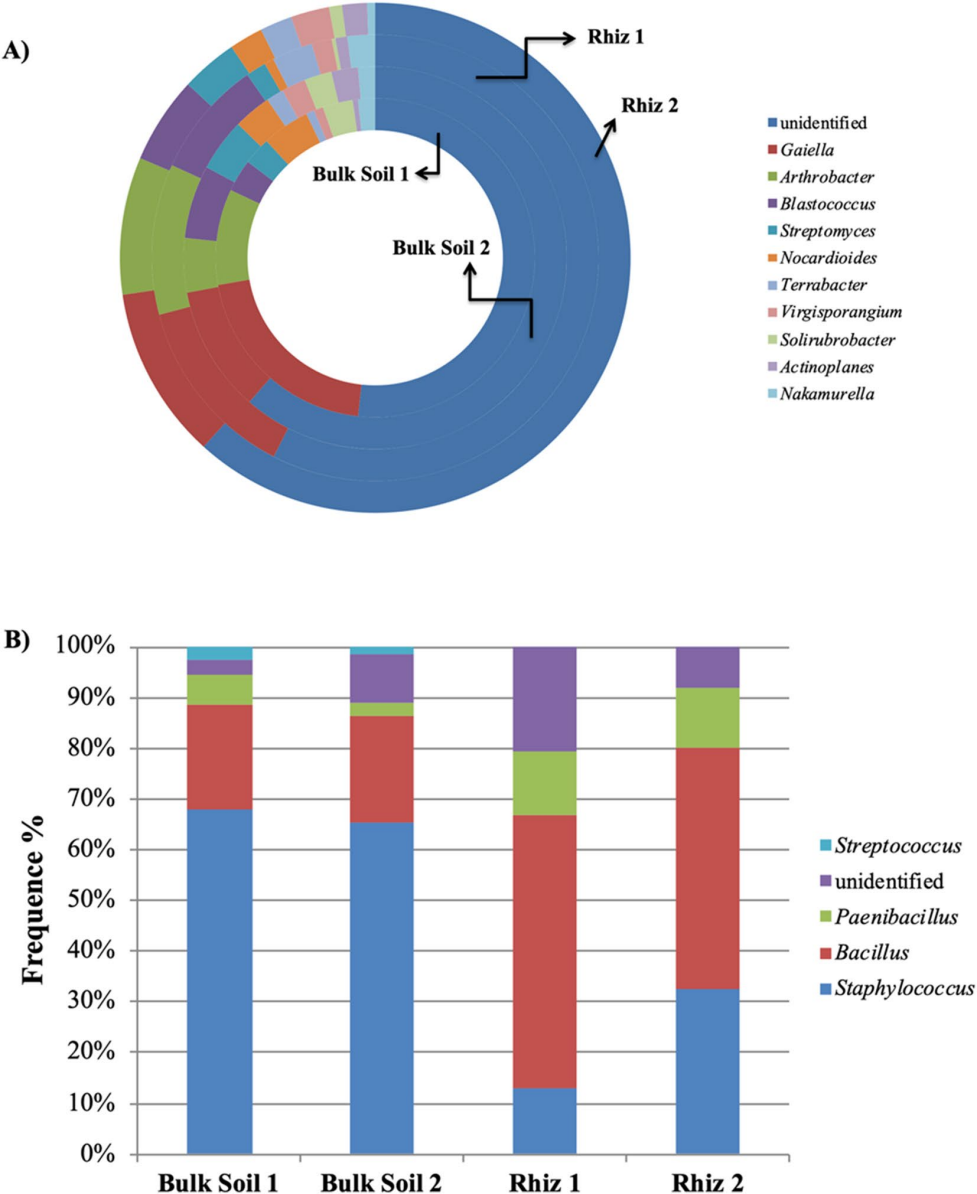
Distribution of the genera belonging to the class (**A**) Actinobacteria and (**B**) Bacilli, in bulk soil and rhizosphere of *V. vinifera* cv. Pinot Noir during the two sampling times (flowering and early fruiting stages). From the center to the edge Bulk Soil 1, Bulk Soil 2, Rhiz 1, Rhiz 2.

**Figure 7:**
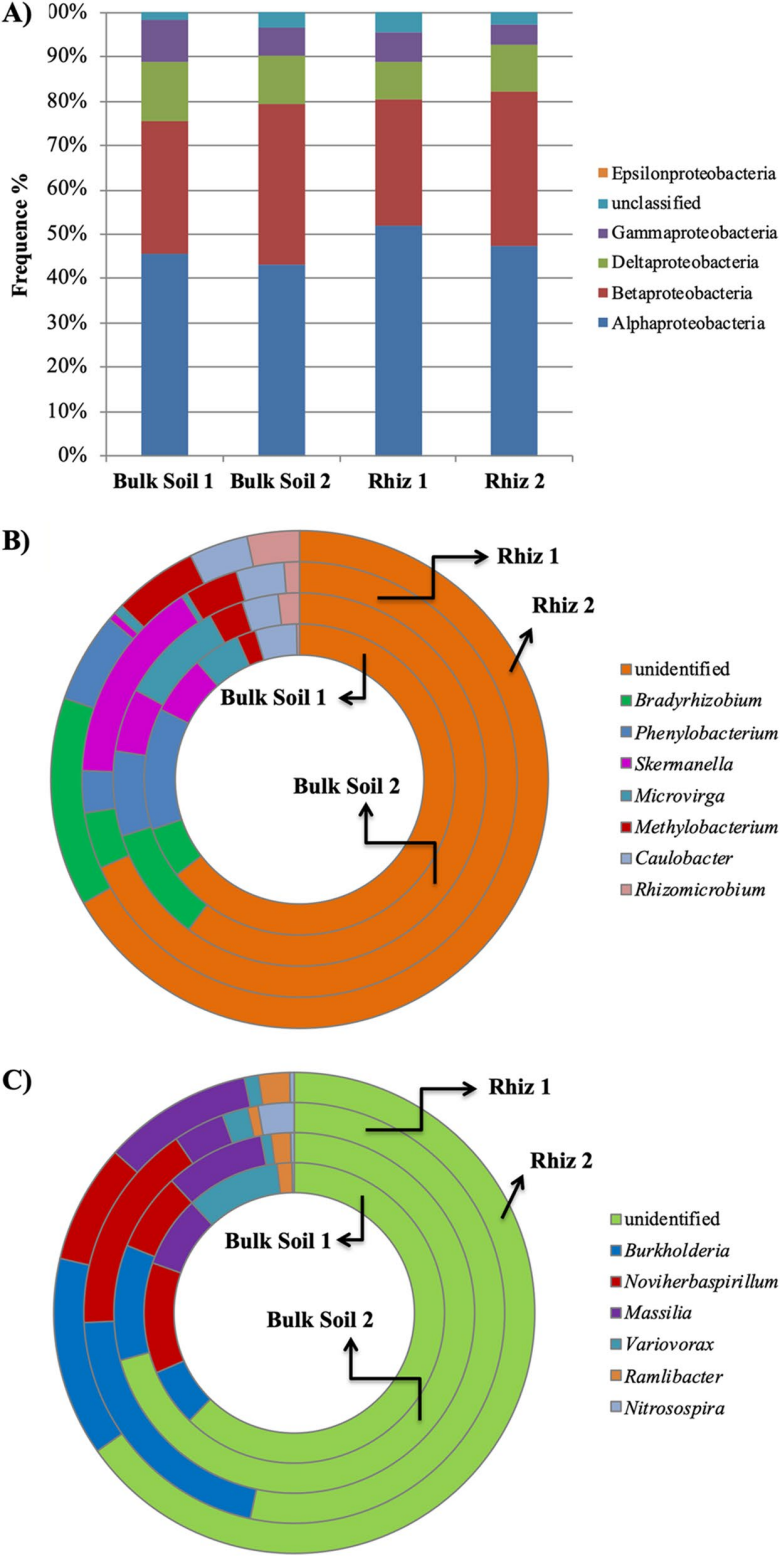
Distribution of (**A**) the different classes of Proteobacteria; (**B**) the genera belonging to the class α-Proteobacteria and (**C**) the genera belonging to the class β-Proteobacteria in bulk soil and rhizosphere of *V. vinifera* cv. Pinot Noir at the two sampling dates (flowering and early fruiting stages). From the centre to the edge Bulk Soil 1, Bulk Soil 2, Rhiz 1, Rhiz 2.

The original Article has been corrected.

