# Supplementary material for: Discovering the bacteriome of Vitis vinifera cv. Pinot Noir in a conventionally managed vineyard
Source: Sci Rep. 2020 Apr 15;10:6453. doi: 10.1038/s41598-020-63154-w (PMC7160115; doi:10.1038/s41598-020-63154-w)
Supplement: Supplementary file 1 — Supplementary Information. [file 41598_2020_63154_MOESM1_ESM.pdf]

Discovering the bacteriome of *Vitis vinifera* cv. Pinot Noir in a conventionally managed vineyard

Elisa Gamalero[1], Elisa Bona[2], Giorgia Novello[1], Lara Boatti[3], Flavio Mignone[1,3], Nadia Massa[1], Patrizia Cesaro[1*], Graziella Berta[1], Guido Lingua[1]

[1] Università del Piemonte Orientale, Dipartimento di Scienze e Innovazione Tecnologica, viale T. Michel 11, Alessandria 15121, Italy

[2] Università del Piemonte Orientale, Dipartimento di Scienze e Innovazione Tecnologica, Piazza San Eusebio 5, 13100 Vercelli, Italy

[3] SmartSeq s.r.l., spin-off of the Università del Piemonte Orientale, Viale T. Michel 11, Alessandria 15121, Italy

Corresponding author:

Dipartimento di Scienze e Innovazione Tecnologica, Università del Piemonte Orientale, Viale T. Michel 11, Alessandria 15121, Italy

Tel: 0131 360 244

Fax: 0131 360 243

E-mail: patrizia.cesaro@uniupo.it

Table S1. Full list of the bacterial species identified in the conventional pest management vineyard and their frequencies and significancies.

phylum	class	order	genus	species	Frequencies				Significancies			
					BS1	BS2	Rhiz1	Rhiz2	BS1 vs BS2	BS1 vs Rhiz1	BS2 Vs Rhiz2	Rhiz1 vs Rhiz2
Actinobacteria	Actinobacteria	Actinomycetales	denovo	denovo	8,49	7,18	16,9	8,75	0,222	0,056	0,151	0,841
Actinobacteria	Actinobacteria	Actinomycetales	denovo	denovo	4,95	7,92	7,57	5,56	0,032	0,151	0,548	0,690
Actinobacteria	Actinobacteria	Solirubrobacterales	denovo	denovo	4,46	3,66	2,98	3,82	0,690	0,841	0,421	0,095
Actinobacteria	Actinobacteria	denovo	denovo	denovo	3,88	2,02	2,89	2,01	1,000	0,841	0,421	0,249
Gemmatimonadetes	Gemmatimonadetes	Gemmatimonadales	Gemmatimonas	Gemmatimonadaceae bacterium LWQ133	0,44	3,36	0,83	2,47	0,094	0,753	0,690	0,008
Proteobacteria	Alphaproteobacteria	Rhizobiales	denovo	denovo	2,09	1,94	2,51	1,62	0,008	0,310	0,548	0,310
Actinobacteria	Actinobacteria	Gaiellales	Gaiella	bacterium Ellin6526	2,41	1,33	2,81	1,77	0,841	0,690	0,095	1,000
Proteobacteria	Betaproteobacteria	denovo	denovo	denovo	0,64	2,42	1,08	2,56	0,056	0,841	0,841	0,008
Actinobacteria	Actinobacteria	Actinomycetales	denovo	denovo	0,61	2,36	1,53	2,23	0,690	0,421	0,310	0,421
Actinobacteria	Actinobacteria	Actinomycetales	denovo	denovo	0,78	0,99	4,48	1,52	0,142	0,008	0,021	0,095
Proteobacteria	Alphaproteobacteria	Rhizobiales	denovo	denovo	0,58	2,18	0,82	2,00	0,016	0,841	0,753	0,008
Proteobacteria	Alphaproteobacteria	Rhodospirillales	denovo	denovo	1,31	1,07	1,69	1,93	0,173	0,222	0,016	0,222
Proteobacteria	Deltaproteobacteria	Myxococcales	denovo	denovo	1,26	1,30	1,24	1,80	0,222	0,690	0,548	0,095
Proteobacteria	Betaproteobacteria	Burkholderiales	denovo	denovo	0,55	2,16	0,69	1,64	0,180	1,000	0,180	1,000
Actinobacteria	Actinobacteria	Gaiellales	Gaiella	bacterium Ellin7530	0,61	1,13	2,25	1,52	0,095	0,462	0,295	0,059

Actinobacteria	Actinobacteria	Actinomycetales	Blastococcus	bacterium Ellin6023	0,88	1,14	2,12	1,36	0,690	0,690	0,310	0,841
Actinobacteria	Actinobacteria	Acidimicrobiales	denovo	denovo	2,81	1,11	1,13	0,81	0,310	1,000	0,530	0,059
Actinobacteria	Actinobacteria	Actinomycetales	denovo	denovo	2,52	1,07	0,87	1,12	0,548	0,600	0,310	0,548
Proteobacteria	Betaproteobacteria	Burkholderiales	denovo	denovo	1,48	1,90	0,43	1,09	0,690	0,841	0,249	0,151
Proteobacteria	Alphaproteobacteria	Rhodospirillales	denovo	denovo	1,27	1,22	1,06	1,24	0,095	0,753	0,222	0,463
Actinobacteria	Actinobacteria	Gaiellales	Gaiella	agricultural soil bacterium SC-I92	2,28	0,81	1,29	0,81	0,841	0,421	0,690	0,095
Firmicutes	Bacilli	Bacillales	Staphylococcus	Staphylococcus epidermidis	5,76	0,59	0,03	0,05	0,526	0,518	0,248	0,528
Actinobacteria	denovo	denovo	denovo	denovo	1,04	1,03	1,43	1,04	0,056	0,095	0,095	0,056
Proteobacteria	denovo	denovo	denovo	denovo	0,59	1,26	1,40	0,91	0,310	0,548	0,421	0,310
Gemmatimonadetes	Gemmatimonadetes	Gemmatimonadales	Gemmatimonas	Gemmatimonadetes bacterium KBS708	0,29	1,38	0,35	1,34	0,151	0,548	0,421	0,222
Gemmatimonadetes	Gemmatimonadetes	Gemmatimonadales	Gemmatimonas	unidentified bacterium	0,44	0,95	0,89	1,30	0,421	0,841	0,421	0,095
Proteobacteria	Alphaproteobacteria	denovo	denovo	denovo	0,88	0,83	1,28	0,99	0,310	1,000	1,000	0,295
Actinobacteria	Actinobacteria	Actinomycetales	denovo	denovo	0,57	0,94	1,09	1,13	0,530	0,841	0,462	0,463
Actinobacteria	Actinobacteria	Actinomycetales	denovo	denovo	1,58	0,50	1,29	0,73	0,032	1,000	0,690	0,047
Acidobacteria	Acidobacteria_Gp1	denovo	denovo	denovo	0,16	0,55	0,56	1,69	0,690	0,310	0,548	0,690
Proteobacteria	Betaproteobacteria	Burkholderiales	denovo	denovo	0,64	1,01	0,63	0,99	0,340	0,012	0,151	0,841
Proteobacteria	Betaproteobacteria	Burkholderiales	denovo	denovo	1,26	0,99	0,64	0,57	0,032	0,528	0,421	0,008
Actinobacteria	Actinobacteria	Actinomycetales	Blastococcus	Blastococcus sp. OS1-29	0,34	0,61	1,42	0,76	0,916	0,917	0,462	0,421
Proteobacteria	Deltaproteobacteria	Myxococcales	denovo	denovo	0,80	0,79	0,69	0,70	1,000	1,000	0,346	0,151

Actinobacteria	Actinobacteria	Actinomycetales	denovo	denovo	0,03	0,21	1,62	1,03	0,293	0,421	0,209	0,834
Firmicutes	Bacilli	Bacillales	Staphylococcus	Staphylococcus epidermidis PM221	3,54	0,39	0,05	0,06	0,222	0,095	0,310	0,753
Actinobacteria	Actinobacteria	Gaiellales	Gaiella	bacterium Ellin7545	0,83	0,60	0,76	0,69	0,402	0,295	0,753	0,834
Bacteroidetes	Sphingobacteriia	Sphingobacteriales	denovo	denovo	1,03	0,43	0,37	0,81	0,690	0,690	0,421	0,222
Actinobacteria	Actinobacteria	Actinomycetales	Kineosporia	Kineosporia rhamnosa (T)	1,45	0,68	0,17	0,33	0,548	0,032	0,094	0,295
Acidobacteria	Acidobacteria_Gp3	Gp3	Gp3	unidentified bacterium	0,17	0,33	0,25	1,20	1,000	0,032	0,032	0,841
Actinobacteria	Actinobacteria	Actinomycetales	Arthrobacter	Arthrobacter sp. L13	0,13	0,05	1,37	0,82	1,000	0,090	0,054	0,402
Acidobacteria	Acidobacteria_Gp3	Gp3	Gp3	bacterium enrichment culture clone SC-2_22	0,00	0,71	0,21	0,81	0,012	0,753	0,674	0,095
Gemmatimonadetes	Gemmatimonadetes	Gemmatimonadales	Gemmatimonas	Microcystis sp. SAG 43.90	0,03	0,78	0,20	0,61	0,056	0,833	0,753	0,012
Actinobacteria	Actinobacteria	Actinomycetales	Arthrobacter	Arthrobacter sp. S88	0,38	0,34	0,90	0,38	0,295	0,829	1,000	0,021
Chloroflexi	Ktedonobacteria	Ktedonobacterales	denovo	denovo	0,07	0,00	1,52	0,46	0,034	0,139	0,310	0,095
Actinobacteria	Actinobacteria	Actinomycetales	denovo	denovo	0,35	0,51	0,38	0,39	0,046	0,112	0,047	0,008
Gemmatimonadetes	Gemmatimonadetes	Gemmatimonadales	Gemmatimonas	Gemmatimonadetes bacterium Ellin7146	0,32	0,40	0,17	0,62	0,139	0,139	0,421	0,056
Proteobacteria	Gammaproteobacteria	Xanthomonadales	denovo	denovo	0,21	0,63	0,15	0,47	0,095	0,753	1,000	0,151
Proteobacteria	Alphaproteobacteria	Rhizobiales	Bradyrhizobium	Bradyrhizobium sp. WSM471	0,35	0,57	0,07	0,49	0,151	0,344	0,917	0,675
Acidobacteria	Acidobacteria_Gp1	Gp1	Gp1	bacterium Ellin6528	0,03	0,37	0,13	0,72	1,000	0,690	0,095	0,151
Gemmatimonadetes	Gemmatimonadetes	Gemmatimonadales	Gemmatimonas	bacterium Ellin5290	0,02	0,49	0,33	0,49	0,834	0,115	0,295	0,172
Actinobacteria	Actinobacteria	Actinomycetales	denovo	denovo	0,44	0,63	0,26	0,20	0,172	0,656	0,094	0,746
Gemmatimonadetes	Gemmatimonadetes	Gemmatimonadales	Gemmatimonas	bacterium enrichment culture clone auto9_4W	0,03	0,31	0,17	0,72	0,031	0,052	0,834	0,548

Actinobacteria	Actinobacteria	Actinomycetales	Nakamurella	Humicoccus sp. L1886	0,50	0,28	0,94	0,08	0,021	1,000	0,753	0,008
Actinobacteria	Actinobacteria	Gaiellales	Gaiella	Gaiella occulta (T)	1,16	0,36	0,14	0,13	0,402	0,829	0,463	1,000
Firmicutes	Bacilli	Bacillales	Staphylococcus	Staphylococcus epidermidis RP62A	1,54	0,28	0,00	0,02	0,526	0,239	0,168	0,480
Proteobacteria	Betaproteobacteria	Burkholderiales	Noviherbaspirillum	Comamonadaceae bacterium MSCB-9	0,00	0,32	0,60	0,33	0,249	0,141	0,095	0,093
Actinobacteria	Actinobacteria	Solirubrobacterales	Conexibacter	bacterium Ellin504	0,22	0,18	0,46	0,39	0,548	0,075	0,690	0,095
Proteobacteria	Gammaproteobacteria	denovo	denovo	denovo	0,16	0,40	0,40	0,25	0,036	0,340	0,310	0,008
Acidobacteria	Acidobacteria_Gp3	denovo	denovo	denovo	0,00	0,59	0,01	0,32	0,690	0,346	0,222	0,421
Actinobacteria	Actinobacteria	Actinomycetales	Blastococcus	Blastococcus sp. L1961	0,76	0,20	0,13	0,17	0,249	0,462	0,834	0,666
Proteobacteria	Betaproteobacteria	Burkholderiales	Burkholderia	Burkholderia sp. MS4t	0,00	0,41	0,23	0,27	1,000	0,670	0,141	0,525
Actinobacteria	Actinobacteria	Actinomycetales	Marmoricola	Marmoricola sp. Gsoil 818	0,50	0,27	0,20	0,17	0,016	0,116	0,600	0,841
Proteobacteria	Alphaproteobacteria	Rhizobiales	denovo	denovo	0,12	0,23	0,03	0,47	0,310	0,750	0,151	0,059
Actinobacteria	Actinobacteria	Actinomycetales	Terrabacter	Terrabacter lapilli (T)	0,02	0,11	0,69	0,21	0,753	0,548	0,248	0,458
Actinobacteria	Actinobacteria	Actinomycetales	Blastococcus	Blastococcus aggregatus	0,53	0,28	0,16	0,11	0,424	0,045	0,025	0,140
Proteobacteria	Alphaproteobacteria	Rhodospirillales	denovo	denovo	0,12	0,24	0,38	0,21	0,052	0,180	0,094	0,059
Proteobacteria	Betaproteobacteria	Burkholderiales	Noviherbaspirillum	Herbaspirillum sp. Sco-D20	0,10	0,26	0,36	0,19	0,310	0,458	0,841	0,115
Actinobacteria	Actinobacteria	Solirubrobacterales	Conexibacter	Rubrobacterineae bacterium BR7-21	0,13	0,31	0,19	0,21	0,195	0,084	0,047	0,690
Acidobacteria	Acidobacteria_Gp3	Gp3	Gp3	Acidobacteria bacterium SCGC AAA204-D14	0,00	0,09	0,08	0,51	0,027	0,829	0,012	0,012
Actinobacteria	Actinobacteria	Solirubrobacterales	Solirubrobacter	bacterium Ellin6048	0,41	0,19	0,09	0,22	1,000	0,518	0,753	0,344
Actinobacteria	Actinobacteria	Actinomycetales	Nocardioides	bacterium Ellin6014	0,39	0,21	0,10	0,21	0,753	0,458	0,338	0,504

Actinobacteria	Actinobacteria	Actinomycetales	Arthrobacter	Arthrobacter sp. SaCR11	0,03	0,09	0,49	0,25	0,396	0,057	0,012	0,346
Acidobacteria	Acidobacteria_Gp1	Gp1	Gp1	Actinomycetales	0,16	0,18	0,10	0,31	0,295	0,402	0,463	0,141
Actinobacteria	Actinobacteria	Actinomycetales	Blastococcus	Candidatus Blastococcus massiliensis AP3	0,26	0,06	0,29	0,26	0,090	0,289	0,690	0,249
Proteobacteria	Deltaproteobacteria	Myxococcales	denovo	denovo	0,00	0,23	0,17	0,27	0,205	0,834	0,057	0,209
Actinobacteria	Actinobacteria	Actinomycetales	Arthrobacter	Arthrobacter sp. HS4-1	0,45	0,06	0,33	0,11	0,014	0,054	0,036	0,074
Proteobacteria	Alphaproteobacteria	Rhizobiales	Methylobacterium	Methylobacterium sp. MG- 2011-64-DP	0,00	0,22	0,13	0,27	0,528	0,596	0,248	0,461
Actinobacteria	Actinobacteria	Acidimicrobiales	Iamia	bacterium Ellin5273	0,51	0,17	0,08	0,10	0,441	0,906	0,424	0,180
Actinobacteria	Actinobacteria	Actinomycetales	Nakamurella	Nakamurella flavida	0,16	0,31	0,10	0,12	1,000	0,402	0,202	0,834
Actinobacteria	Actinobacteria	Solirubrobacterales	Solirubrobacter	bacterium Ellin5249	0,59	0,23	0,03	0,03	0,665	0,504	0,823	0,813
Actinobacteria	Actinobacteria	Solirubrobacterales	Solirubrobacter	Solirubrobacter ginsenosidimutans	0,63	0,23	0,02	0,02	0,346	1,000	0,458	0,136
Acidobacteria	Acidobacteria_Gp3	Gp3	Gp3	Actinomycetales	0,00	0,09	0,04	0,42	0,463	0,463	0,917	1,000
Chloroflexi	denovo	denovo	denovo	denovo	0,39	0,14	0,26	0,06	0,295	0,036	0,016	1,000
Acidobacteria	Acidobacteria_Gp1	Gp1	Gp1	bacterium Ellin6547	0,03	0,18	0,12	0,25	0,753	0,344	0,690	0,036
Gemmatimonadetes	Gemmatimonadetes	Gemmatimonadales	Gemmatimonas	Gemmatimonadetes bacterium LX87	0,09	0,26	0,02	0,21	0,829	0,914	0,074	0,142
Actinobacteria	Actinobacteria	Gaiellales	Gaiella	bacterium Ellin7507	0,08	0,17	0,19	0,19	0,401	0,525	0,032	0,141
Actinobacteria	Actinobacteria	Actinomycetales	Intrasporangium	Intrasporangium calvum DSM 43043	0,38	0,26	0,05	0,06	NA	0,180	0,424	0,519
Acidobacteria	Acidobacteria_Gp1	Gp1	Gp1	bacterium Ellin7544	0,13	0,08	0,21	0,24	0,018	0,441	0,530	0,090
Gemmatimonadetes	Gemmatimonadetes	Gemmatimonadales	Gemmatimonas	Gemmatimonas aurantiaca	0,00	0,21	0,03	0,26	0,441	0,724	0,424	0,180
Actinobacteria	Actinobacteria	Gaiellales	Gaiella	unidentified bacterium	0,48	0,06	0,25	0,05	0,753	0,046	0,139	0,284

Proteobacteria	Deltaproteobacteria	Myxococcales	Kofleria	Kofleria flava	0,01	0,17	0,05	0,27	0,448	0,750	0,345	0,234
Actinobacteria	Actinobacteria	Actinomycetales	Arthrobacter	Arthrobacter sp. M29	0,00	0,12	0,26	0,20	0,289	1,000	0,528	0,012
Actinobacteria	Actinobacteria	Actinomycetales	Marmoricola	Nocardioides iriomotensis	0,42	0,16	0,12	0,04	0,917	0,036	0,142	0,584
Actinobacteria	Actinobacteria	Actinomycetales	Arthrobacter	Arthrobacter oryzae	0,48	0,15	0,02	0,08	0,434	0,724	0,194	0,441
Acidobacteria	Acidobacteria_Gp1	Candidatus_Koribacter	Candidatus_Koribacter	Candidatus Koribacter versatilis Ellin345	0,00	0,11	0,12	0,28	0,072	0,746	NA	0,072
Proteobacteria	Alphaproteobacteria	Sphingomonadales	denovo	denovo	0,18	0,24	0,11	0,08	1,000	0,674	0,115	0,753
Proteobacteria	Alphaproteobacteria	Caulobacterales	Phenylobacterium	Phenylobacterium sp. W2.09- 62	0,24	0,13	0,09	0,16	0,396	1,000	0,916	1,000
Proteobacteria	Alphaproteobacteria	Rhizobiales	denovo	denovo	0,06	0,09	0,21	0,20	0,009	0,192	1,000	0,020
Actinobacteria	Actinobacteria	Actinomycetales	Arthrobacter	Arthrobacter sp. 7B-895	0,00	0,03	0,18	0,30	0,015	0,168	0,753	0,462
Nitrospirae	Nitrospira	Nitrospirales	Nitrospira	Candidatus Nitrospira bockiana	0,03	0,06	0,05	0,34	0,205	0,373	0,917	0,753
Proteobacteria	Alphaproteobacteria	Rhodospirillales	Skermanella	Skermanella aerolata	0,28	0,30	0,00	0,02	0,012	0,525	0,690	0,012
Actinobacteria	Actinobacteria	Actinomycetales	Terrabacter	Terrabacter carboxydivorans (T)	0,03	0,03	0,28	0,20	0,138	1,000	0,517	0,401
Actinobacteria	Actinobacteria	Gaiellales	Gaiella	bacterium enrichment culture clone F44x_D7_T3_E05	0,23	0,03	0,37	0,06	0,180	0,441	NA	0,424
Actinobacteria	Actinobacteria	Actinomycetales	Tetrasphaera	Tetrasphaera sp. Ellin115	0,44	0,20	0,03	0,00	0,834	0,600	0,141	0,675
Actinobacteria	Actinobacteria	Actinomycetales	Nocardioides	Nocardioides sp. S147	0,45	0,19	0,00	0,02	0,071	0,434	0,528	1,000
Actinobacteria	Actinobacteria	Actinomycetales	Nocardioides	Nocardioides islandensis (T)	0,16	0,19	0,04	0,13	0,530	0,222	0,292	0,752
Bacteroidetes	Flavobacteriia	Flavobacteriales	Chryseobacterium	Chryseobacterium sp. CI02	0,03	0,37	0,02	0,03	0,290	0,914	0,289	0,670
Acidobacteria	Acidobacteria_Gp1	Gp1	Gp1	bacterium Ellin7522	0,06	0,15	0,10	0,17	0,387	0,737	0,093	0,142
Actinobacteria	Actinobacteria	Actinomycetales	Streptomyces	Streptomyces viridochromogenes subsp. komabensis	0,02	0,25	0,00	0,16	0,441	0,724	0,424	0,180

Proteobacteria	Betaproteobacteria	Burkholderiales	Noviherbaspirillum	Oxalobacter sp. W1.09-142	0,19	0,08	0,12	0,16	0,115	0,113	0,402	0,195
Actinobacteria	Actinobacteria	Actinomycetales	Arthrobacter	Arthrobacter globiformis	0,26	0,13	0,14	0,05	0,750	0,834	0,172	0,173
Proteobacteria	Alphaproteobacteria	Caulobacterales	denovo	denovo	0,18	0,17	0,08	0,08	0,203	0,830	1,000	0,015
Proteobacteria	Alphaproteobacteria	Rhodospirillales	Skermanella	Azospirillum sp. enrichment culture clone VanCtr101	0,09	0,08	0,45	0,00	1,000	0,341	0,070	0,027
Proteobacteria	Alphaproteobacteria	Sphingomonadales	denovo	denovo	0,02	0,17	0,17	0,10	0,084	0,130	0,501	0,576
Acidobacteria	Acidobacteria_Gp3	Gp3	Gp3	bacterium Ellin7504	0,00	0,04	0,05	0,29	0,396	1,000	0,517	0,205
Acidobacteria	Acidobacteria_Gp10	Gp10	Gp10	Acidobacteria bacterium WY11	0,10	0,23	0,05	0,06	1,000	1,000	0,015	0,591
Proteobacteria	Alphaproteobacteria	Rhizobiales	Bradyrhizobium	bacterium Ellin6538	0,00	0,09	0,00	0,25	0,158	0,519	1,000	0,011
Firmicutes	Bacilli	Bacillales	Bacillus	Bacillus sp. SKM136	0,47	0,04	0,07	0,04	0,242	0,290	0,043	0,130
Acidobacteria	Acidobacteria_Gp3	Gp3	Gp3	agricultural soil bacterium SC-I-77	0,06	0,09	0,03	0,20	0,674	1,000	0,093	1,000
Bacteroidetes	Sphingobacteriia	Sphingobacteriales	Segetibacter	Segetibacter aerophilus	0,16	0,09	0,08	0,11	0,527	1,000	0,016	0,239
Actinobacteria	Actinobacteria	Actinomycetales	Motilibacter	Actinomycetales bacterium RS-16	0,00	0,09	0,05	0,19	0,023	0,018	0,075	0,032
Proteobacteria	Betaproteobacteria	Burkholderiales	Ramlibacter	Ramlibacter tataouinensis TTB310	0,09	0,08	0,03	0,17	0,169	0,830	0,058	0,331
Actinobacteria	Actinobacteria	Actinomycetales	denovo	denovo	0,22	0,10	0,07	0,07	0,829	0,265	0,595	0,480
Actinobacteria	Actinobacteria	Actinomycetales	Arthrobacter	Arthrobacter sp. LM3(2011)	0,24	0,09	0,10	0,05	1,000	0,085	0,056	0,478
Actinobacteria	Actinobacteria	Acidimicrobiales	Aciditerrimonas	Acidimicrobidae bacterium Ellin 7143	0,03	0,11	0,16	0,08	0,600	0,230	0,036	0,443
Acidobacteria	Acidobacteria_Gp3	Gp3	Gp3	Acidobacteria bacterium LWQ4	0,09	0,11	0,05	0,12	1,000	0,746	0,245	0,750
Bacteroidetes	denovo	denovo	denovo	denovo	0,23	0,11	0,15	0,00	0,527	1,000	0,599	0,116
Proteobacteria	Deltaproteobacteria	denovo	denovo	denovo	0,21	0,13	0,05	0,05	0,834	1,000	0,173	0,402

Actinobacteria	Actinobacteria	Actinomycetales	Amycolatopsis	Amycolatopsis lexingtonensis (T)	0,16	0,18	0,00	0,05	0,746	0,280	0,264	0,821
Actinobacteria	Actinobacteria	Actinomycetales	Terrabacter	Terrabacter terrae (T)	0,00	0,06	0,11	0,17	0,797	0,345	0,041	0,202
Actinobacteria	Actinobacteria	Acidimicrobiales	Ilumatobacter	unidentified bacterium	0,27	0,09	0,03	0,05	0,065	0,018	0,074	0,112
Proteobacteria	Alphaproteobacteria	Rhodospirillales	Roseomonas	Acetobacteraceae bacterium GIMN 1.017	0,00	0,18	0,10	0,06	0,674	0,106	0,206	0,445
Acidobacteria	Acidobacteria_Gp1	Gp1	Gp1	bacterium Ellin7529	0,00	0,17	0,00	0,12	0,278	0,906	0,398	0,066
Actinobacteria	Actinobacteria	Solirubrobacterales	Solirubrobacter	Solirubrobacter sp. Gsoil 917	0,13	0,12	0,07	0,06	0,295	1,000	0,112	0,747
Firmicutes	Bacilli	Bacillales	denovo	denovo	0,38	0,03	0,12	0,00	0,401	0,280	1,000	0,089
Proteobacteria	Alphaproteobacteria	Caulobacterales	Caulobacter	Caulobacter vibrioides	0,00	0,10	0,09	0,12	0,093	0,480	0,462	0,085
Firmicutes	Bacilli	Bacillales	denovo	denovo	0,06	0,18	0,07	0,03	0,519	0,203	0,012	1,000
Actinobacteria	Actinobacteria	Actinomycetales	Actinoplanes	Actinoplanes sp. IR56-Co102	0,00	0,24	0,00	0,04	1,000	0,607	0,192	1,000
Actinobacteria	Actinobacteria	Actinomycetales	Mycobacterium	Mycobacterium sp. N1730C	0,32	0,11	0,01	0,00	1,000	0,525	0,015	0,338
Actinobacteria	Actinobacteria	Actinomycetales	Terrabacter	Terrabacter sp. A2-62	0,16	0,04	0,10	0,09	0,009	1,000	0,399	0,010
Actinobacteria	Actinobacteria	Actinomycetales	Arthrobacter	Arthrobacter sp. CFM 20	0,21	0,07	0,08	0,04	0,667	0,332	0,023	0,462
Actinobacteria	Actinobacteria	Actinomycetales	Streptomyces	Streptomyces sp. 1MR-8	0,00	0,17	0,03	0,08	0,829	0,462	0,289	0,916
Proteobacteria	Betaproteobacteria	Burkholderiales	Burkholderia	Burkholderia sp. MG-2011-1- AC	0,00	0,04	0,09	0,16	0,449	0,666	0,243	0,833
Proteobacteria	Betaproteobacteria	Burkholderiales	Burkholderia	bacterium enrichment culture clone SC-2_86	0,00	0,01	0,21	0,11	0,034	0,734	0,249	0,011
Proteobacteria	Alphaproteobacteria	Rhizobiales	Pseudolabrys	methanogenic prokaryote enrichment culture B19_111	0,03	0,06	0,07	0,14	0,396	0,723	0,009	0,600
Proteobacteria	Alphaproteobacteria	Rhizobiales	denovo	denovo	0,00	0,15	0,07	0,06	0,071	0,331	1,000	0,243
Proteobacteria	Alphaproteobacteria	Caulobacterales	Phenylobacterium	Phenylobacterium sp. P-28	0,02	0,17	0,03	0,06	0,752	0,829	0,172	0,115

Proteobacteria	Alphaproteobacteria	Alphaproteobacteria_incertae_sedis	Rhizomicrobium	bacterium Ellin329	0,03	0,09	0,00	0,14	0,584	0,502	0,034	0,209
Actinobacteria	Actinobacteria	Actinomycetales	Cryptosporangium	Cryptosporangium minutisporangium (T)	0,12	0,17	0,00	0,02	0,066	0,501	0,203	0,012
Proteobacteria	Alphaproteobacteria	Rhizobiales	Bradyrhizobium	Bradyrhizobium sp. IV-102	0,00	0,09	0,00	0,16	0,511	1,000	0,262	0,101
Proteobacteria	Alphaproteobacteria	Rhodospirillales	Skermanella	Skermanella sp. JC5	0,03	0,18	0,04	0,03	0,236	0,034	0,463	0,675
Actinobacteria	Actinobacteria	Actinomycetales	Tetrasphaera	actinomycete S23403	0,45	0,02	0,01	0,00	0,007	0,029	0,424	0,600
Proteobacteria	Alphaproteobacteria	Alphaproteobacteria_incertae_sedis	Rhizomicrobium	bacterium Ellin335	0,00	0,07	0,03	0,14	0,249	0,236	0,018	0,515
Proteobacteria	Deltaproteobacteria	Myxococcales	Kofleria	Kofleria sp. DSM 53797	0,03	0,07	0,03	0,13	0,044	0,911	0,116	0,091
Actinobacteria	Actinobacteria	Actinomycetales	Nocardioides	Nocardioides sp. S23405	0,17	0,11	0,00	0,05	0,478	0,406	0,246	0,141
Proteobacteria	Betaproteobacteria	Burkholderiales	Massilia	Kartchner Caverns bacterium MI-10a	0,00	0,11	0,00	0,13	0,180	0,180	NA	NA
Actinobacteria	Actinobacteria	Actinomycetales	Actinoplanes	Actinoplanes sp. RI44-Va104	0,00	0,11	0,02	0,11	0,283	0,434	0,114	0,675
Actinobacteria	Actinobacteria	Gaiellales	Gaiella	metal-contaminated soil clone K20-25	0,11	0,18	0,01	0,00	1,000	1,000	1,000	0,833
Actinobacteria	Actinobacteria	Solirubrobacterales	Solirubrobacter	Solirubrobacter soli (T)	0,15	0,14	0,01	0,02	1,000	0,195	0,289	0,371
Actinobacteria	Actinobacteria	Actinomycetales	Streptomyces	Streptomyces canu	0,29	0,08	0,01	0,02	0,009	0,232	0,598	0,059
Actinobacteria	Actinobacteria	Gaiellales	Gaiella	Actinomycetales	0,09	0,01	0,23	0,03	1,000	0,284	0,675	0,168
Proteobacteria	Alphaproteobacteria	Caulobacterales	Phenylobacterium	Phenylobacterium sp. DLS-83	0,09	0,09	0,07	0,05	0,071	0,180	1,000	0,020
Actinobacteria	Actinobacteria	Acidimicrobiales	Aciditerrimonas	Aciditerrimonas ferrireducens (T)	0,03	0,07	0,03	0,11	0,025	0,118	NA	0,424
Actinobacteria	Actinobacteria	Actinomycetales	Amycolatopsis	Amycolatopsis rifamycinica (T)	0,06	0,11	0,05	0,06	0,670	0,478	0,134	0,797
Actinobacteria	Actinobacteria	Actinomycetales	Streptomyces	Streptomyces sp. NEAU-CF1	0,06	0,09	0,07	0,06	0,915	0,823	0,525	0,338
Acidobacteria	Acidobacteria_Gp1	Gp1	Gp1	Acidobacteriaceae bacterium A2-1c	0,00	0,09	0,03	0,11	0,239	0,023	0,027	0,753

Proteobacteria	Betaproteobacteria	Burkholderiales	Massilia	Massilia aerilata	0,06	0,09	0,00	0,09	0,737	0,737	0,737	0,504
Actinobacteria	Actinobacteria	Actinomycetales	Arthrobacter	Pseudomonas moraviensis	0,03	0,00	0,08	0,14	0,180	0,441	NA	0,424
Actinobacteria	Actinobacteria	Actinomycetales	Arthrobacter	Arthrobacter defluvii	0,05	0,03	0,07	0,11	0,072	0,239	NA	0,424
Acidobacteria	Acidobacteria_Gp1	Gp1	Gp1	bacterium K-5b2	0,00	0,03	0,05	0,14	0,130	0,813	0,346	0,055
Actinobacteria	Actinobacteria	Actinomycetales	Amycolatopsis	Amycolatopsis bullii	0,06	0,10	0,07	0,03	0,345	0,441	0,138	0,112
Proteobacteria	Alphaproteobacteria	Rhizobiales	Methylobacterium	Methylobacterium longum	0,08	0,04	0,07	0,08	0,138	0,827	0,824	0,202
Actinobacteria	Actinobacteria	Actinomycetales	Arthrobacter	soil bacterium 14V-14	0,00	0,04	0,10	0,09	0,171	0,914	0,110	0,434
Gemmatimonadetes	Gemmatimonadetes	Gemmatimonadales	Gemmatimonas	Gemmatimonadetes bacterium WWH24	0,00	0,09	0,00	0,11	0,329	0,140	0,337	0,596
Proteobacteria	Alphaproteobacteria	Caulobacterales	Phenylobacterium	bacterium Ellin5060	0,03	0,09	0,02	0,08	0,344	0,057	1,000	0,596
Actinobacteria	Actinobacteria	Acidimicrobiales	Ilumatobacter	unidentified marine bacterioplankton	0,25	0,04	0,03	0,01	0,398	0,441	0,007	0,424
Proteobacteria	Alphaproteobacteria	Rhizobiales	Beijerinckia	Beijerinckia mobilis (T)	0,00	0,09	0,00	0,11	0,745	0,504	0,825	0,742
Acidobacteria	Acidobacteria_Gp1	Gp1	Gp1	bacterium enrichment culture clone PA_42	0,00	0,11	0,01	0,08	0,528	0,829	0,071	1,000
Actinobacteria	Actinobacteria	Solirubrobacterales	Conexibacter	bacterium Ellin5025	0,02	0,02	0,12	0,09	0,025	0,180	0,246	0,014
Proteobacteria	Alphaproteobacteria	Rhizobiales	Bradyrhizobium	Bradyrhizobium group bacterium Ellin127	0,00	0,09	0,00	0,11	0,130	0,373	0,753	0,456
Proteobacteria	Betaproteobacteria	Burkholderiales	Ramlibacter	Variovorax sp. P-9	0,03	0,07	0,03	0,09	0,607	0,441	0,424	0,424
Actinobacteria	Actinobacteria	Actinomycetales	Terrabacter	Terrabacter sp. Ellin102	0,09	0,06	0,05	0,06	0,106	0,504	0,332	0,386
Firmicutes	Bacilli	Bacillales	Bacillus	Bacillus sp. SKM7	0,23	0,06	0,03	0,02	0,287	0,346	0,242	0,091
Proteobacteria	Alphaproteobacteria	Rhodospirillales	Dongia	bacterium Ellin314	0,08	0,09	0,00	0,06	0,833	0,265	0,045	0,441
Actinobacteria	Actinobacteria	Actinomycetales	Arthrobacter	soil bacterium 14V-08	0,03	0,10	0,07	0,05	0,020	1,000	0,916	0,011

Figure S1. Rarefaction curves for each sample (Bulk Soil 1, Bulk Soil 2, Rhiz 1, Rhiz 2; five sub-samples each). Analysis was performed using MicrobiomeAnalyst, a free available on-line software (https://www.microbiomeanalyst.ca).

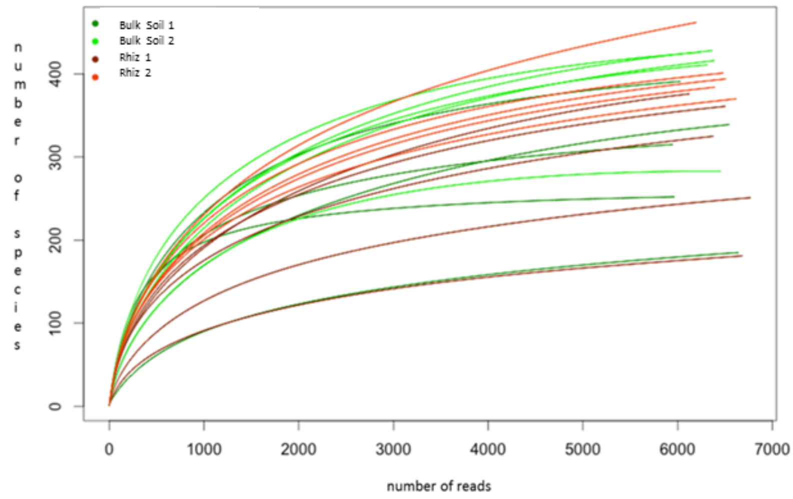

Figure S2. Hierarchical clustering and Heatmap visualization at the genus level measured as Euclidean distance by using the Ward algorithm according to A) variable time (flowering vs. early fruiting time) and B) variable space (bulk soil vs. rhizosphere). Analysis was performed using MicrobiomeAnalyst, a free available on-line software (https://www.microbiomeanalyst.ca).

A)

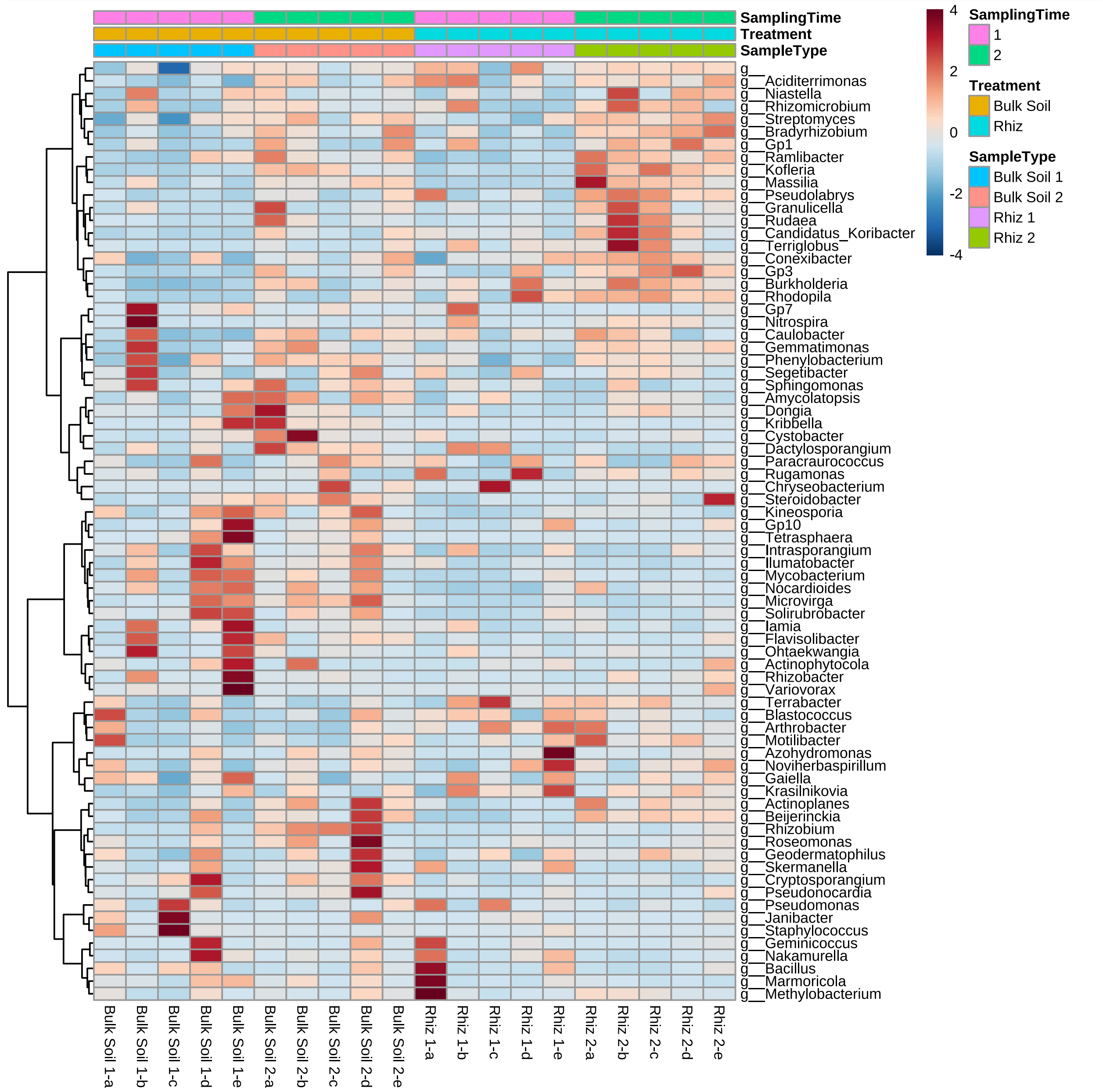

SamplingTime
Treatment
SampleType
4
2
0
-2
-4
SamplingTime
1
2
Treatment
Bulk Soil
Rhiz
SampleType
Bulk Soil 1
Bulk Soil 2
Rhiz 1
Rhiz 2
g__
g__Aciditerrimonas
g__Niastella
g__Rhizomicrobium
g__Streptomyces
g__Bradyrhizobium
g__Gp1
g__Ramlibacter
g__Kofleria
g__Massilia
g__Pseudolabrys
g__Granulicella
g__Rudaea
g__Candidatus_Koribacter
g__Terriglobus
g__Conexibacter
g__Gp3
g__Burkholderia
g__Rhodopila
g__Gp7
g__Nitrospira
g__Caulobacter
g__Gemmatimonas
g__Phenylobacterium
g__Segetibacter
g__Sphingomonas
g__Amycolatopsis
g__Dongia
g__Kribbella
g__Cystobacter
g__Dactylosporangium
g__Paracraurococcus
g__Rugamonas
g__Chryseobacterium
g__Steroidobacter
g__Kineosporia
g__Gp10
g__Tetrasphaera
g__Intrasporangium
g__Ilumatobacter
g__Mycobacterium
g__Nocardioides
g__Microvirga
g__Solirubrobacter
g__Iamia
g__Flavisolibacter
g__Ohtaekwangia
g__Actinophytocola
g__Rhizobacter
g__Variovorax
g__Terrabacter
g__Blastococcus
g__Arthrobacter
g__Motilibacter
g__Azohydromonas
g__Noviherbaspirillum
g__Gaiella
g__Krasilnikovia
g__Actinoplanes
g__Beijerinckia
g__Rhizobium
g__Roseomonas
g__Geodermatophilus
g__Skermanella
g__Cryptosporangium
g__Pseudonocardia
g__Pseudomonas
g__Janibacter
g__Staphylococcus
g__Geminicoccus
g__Nakamurella
g__Bacillus
g__Marmoricola
g__Methylobacterium
Bulk Soil 1-a
Bulk Soil 1-b
Bulk Soil 1-c
Bulk Soil 1-d
Bulk Soil 1-e
Bulk Soil 2-a
Bulk Soil 2-b
Bulk Soil 2-c
Bulk Soil 2-d
Bulk Soil 2-e
Rhiz 1-a
Rhiz 1-b
Rhiz 1-c
Rhiz 1-d
Rhiz 1-e
Rhiz 2-a
Rhiz 2-b
Rhiz 2-c
Rhiz 2-d
Rhiz 2-e

B)

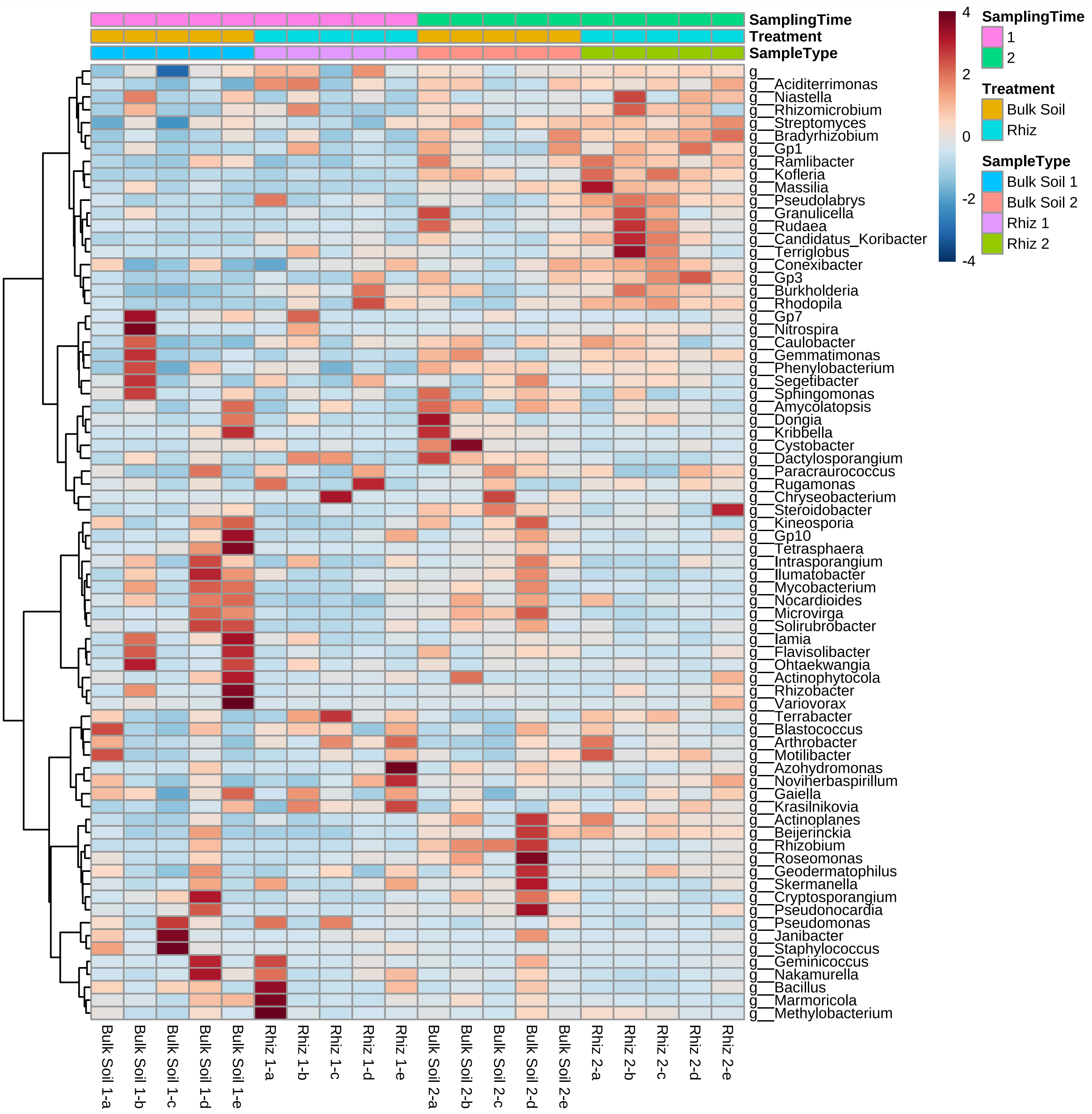

Figure S3. Pattern Search using as variable the sampling time, based on the top 25 genera identified: *Pseudomonas, Nakamurella, Bacillus* and GP7 are the genus more related to the variation of the microbiota according to sampling time. Bars represent positive (pink) and negative (light blue) value of correlation. Analysis was performed using MicrobiomeAnalyst, a free available on-line software (https://www.microbiomeanalyst.ca).

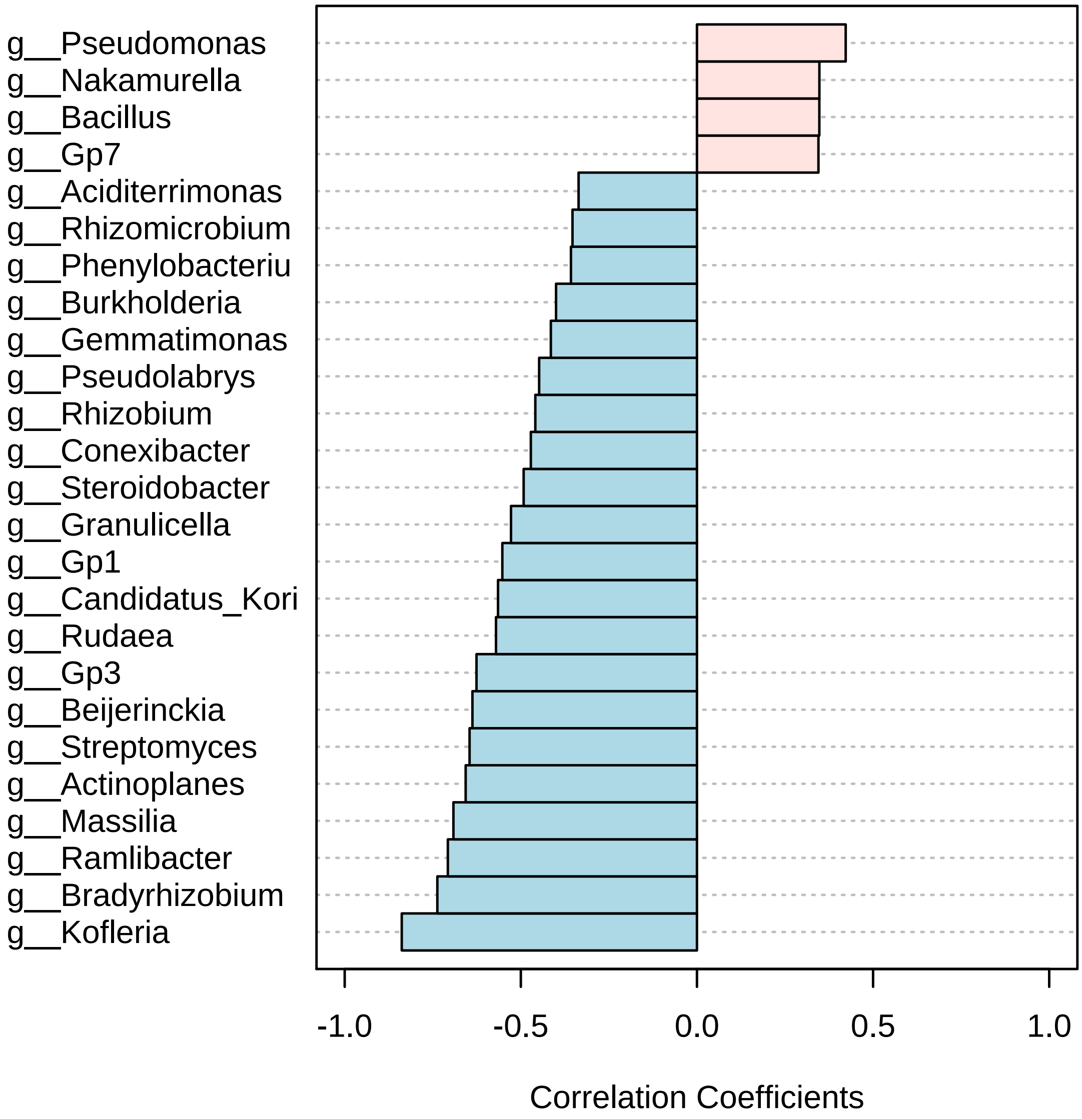
g__Pseudomonas
g__Nakamurella
g__Bacillus
g__Gp7
g__Aciditerrimonas
g__Rhizomicrobium
g__Phenylobacteriu
g__Burkholderia
g__Gemmatimonas
g__Pseudolabrys
g__Rhizobium
g__Conexibacter
g__Steroidobacter
g__Granulicella
g__Gp1
g__Candidatus_Kori
g__Rudaea
g__Gp3
g__Beijerinckia
g__Streptomyces
g__Actinoplanes
g__Massilia
g__Ramlibacter
g__Bradyrhizobium
g__Kofleria
-1.0
-0.5
0.0
0.5
1.0
Correlation Coefficients
